# Targeting epithelial IFN-I signaling restores barrier integrity in anti-TNF refractory Crohn’s disease

**DOI:** 10.1093/ecco-jcc/jjag140

**Published:** 2026-09-19

**Authors:** Safina Gadeock, Mikayla King, D Brent Polk, Andrew J Highton, Shujun Fan, Claudia Campbell, Michael Schultz, Roslyn A Kemp

**Affiliations:** Department of Microbiology and Immunology, Division of Health Sciences, University of Otago, Dunedin, Otago, New Zealand; Division of Pediatric Gastroenterology and Nutrition, The Saban Research Institute, Children’s Hospital Los Angeles, Los Angeles, CA, United States; Department of Microbiology and Immunology, Division of Health Sciences, University of Otago, Dunedin, Otago, New Zealand; Division of Pediatric Gastroenterology and Nutrition, The Saban Research Institute, Children’s Hospital Los Angeles, Los Angeles, CA, United States; Department of Pediatrics, School of Medicine, University of California San Diego, La Jolla, CA, United States; Department of Microbiology and Immunology, Division of Health Sciences, University of Otago, Dunedin, Otago, New Zealand; Department of Microbiology and Immunology, Division of Health Sciences, University of Otago, Dunedin, Otago, New Zealand; Department of Medicine, Division of Health Sciences, University of Otago, Dunedin, Otago, New Zealand; Department of Medicine, Gastroenterology, Health New Zealand, Dunedin, Otago, New Zealand; Department of Medicine, Division of Health Sciences, University of Otago, Dunedin, Otago, New Zealand; Department of Medicine, Gastroenterology, Health New Zealand, Dunedin, Otago, New Zealand; Department of Microbiology and Immunology, Division of Health Sciences, University of Otago, Dunedin, Otago, New Zealand

**Keywords:** interferons, patient-derived organoids, IBD precision medicine

## Abstract

**Background and Aims:**

Type I interferons (IFN-Is) are central regulators of intestinal epithelial homeostasis, yet their role in driving epithelial dysfunction and therapeutic non-response in Crohn’s disease (CD) remains poorly defined. Here, we investigated epithelial tumor necrosis factor (TNF)–IFN-I crosstalk as a previously unrecognized contributor to anti-TNF non-response in CD.

**Methods:**

We analyzed human intestinal biopsies, single-cell RNA sequencing data from CD donors, and used murine TNFR1-deficient and patient-derived organoids exposed to inflammatory cytokines and clinically relevant treatments.

**Results:**

Colonic epithelia from anti-TNF non-responders (NR-CD) showed enrichment of IFN-I genes associated with epithelial stress, impaired regeneration, and inflammatory cytokine production. Using patient-derived and murine TNFR1-deficient organoids, we demonstrated that TNF signaling sustains epithelial IFN-I responses linked to immune regulation and antiviral pathways, establishing a mechanistic connection between chronic TNF signaling and epithelial barrier failure. Low-dose butyrate (0.1 mM) suppressed cytokine-induced IFN-I genes and enhanced epithelial turnover in non-IBD and responder CD (R-CD) organoids but did not rescue the apoptosis and defective repair characteristic of NR-CD organoids. Notably, anti-IFN-I antibody treatment selectively attenuated IFN-I signaling and restored epithelial integrity in NR-CD organoids, identifying epithelial IFN-I blockade as a novel therapeutic rescue strategy for anti-TNF refractory disease.

**Conclusions:**

TNF–IFN-I crosstalk drives epithelial dysfunction in CD. Elevated epithelial IFN-I genes distinguish anti-TNF non-responders and underlie differential therapeutic responses. Together, this work supports integration of epithelial IFN-I gene expression and patient-derived *ex vivo* functional testing into treatment decisions for CD patients.

## Graphical Abstract

**Graphical Abstract:**
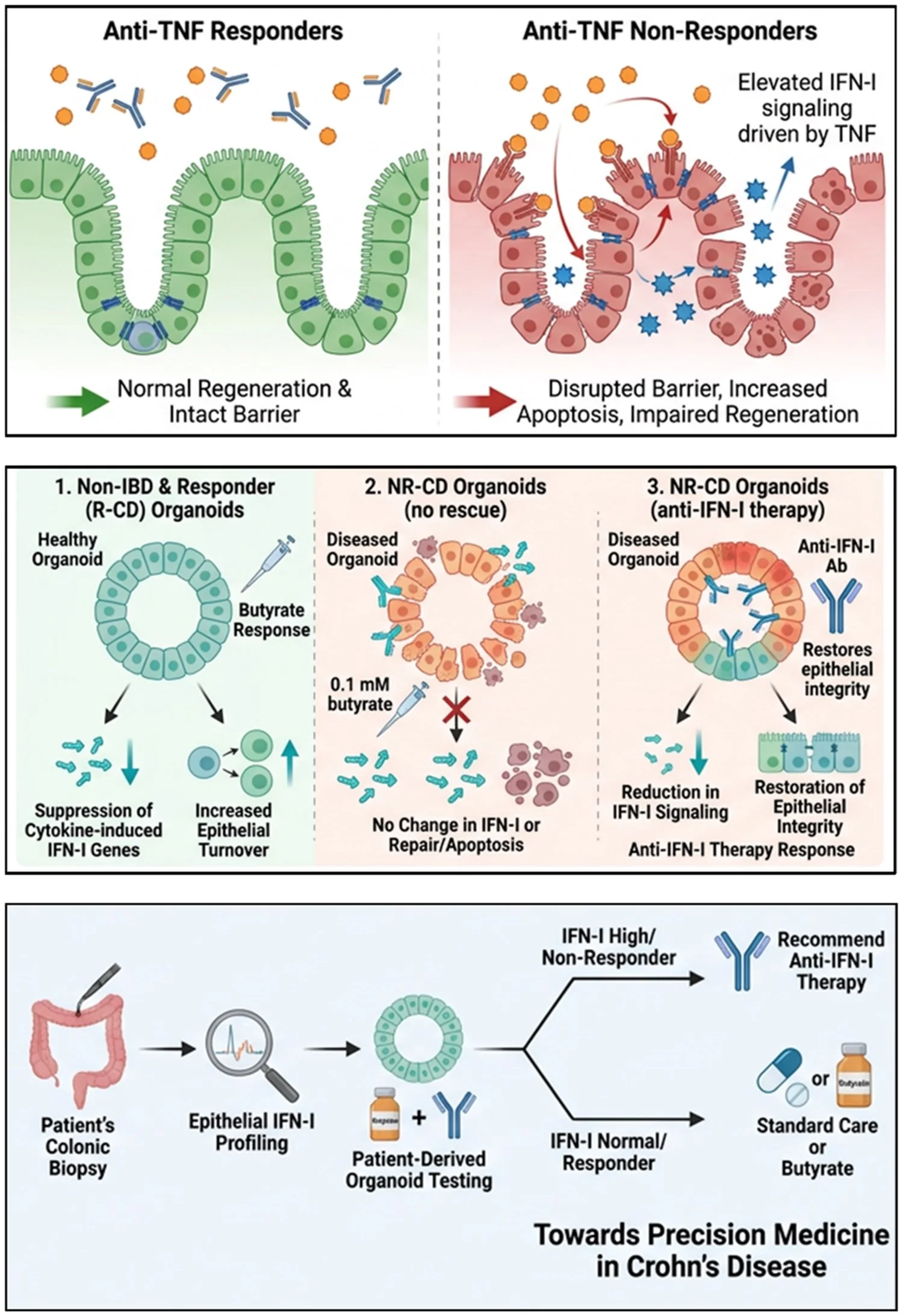

## 1. Introduction

Inflammatory bowel diseases (IBD) are chronic, inflammatory, and relapsing conditions affecting the intestines. IBD treatments that target the immune system, such as anti-tumor necrosis factor (anti-TNF) antibodies, can maintain remission for many patients.^1^ However, 30%-50% of patients are non-responsive to these treatments and experience significant side effects.^1^ IBD anti-TNF “responders” (R; people who respond to anti-TNF antibodies) have a higher abundance of the gut bacteria *Faecalibacterium prausnitzii* and its metabolite, butyrate, compared to “non-responders” (NR; people who do not have primary or secondary responses to anti-TNF antibodies).^2^^,^^3^^,^^4^

Butyrate, a short-chain fatty acid produced by gut bacteria, maintains health of intestinal epithelial cells (IECs).^5^ It serves as the primary energy source for IECs, accounting for ∼70% of their energy requirements.^5^ IECs located at the interface between the microbiome and the gut immune system play a central role in immune signaling during both homeostasis and inflammation.^6^ Under normal conditions, the intestinal epithelium responds to butyrate by regulating host-defense pathways that support cell proliferation, survival, and anti-inflammatory responses.^7^^,^^8^

Interferons (IFNs) are central to regulation of host defense in IBD.^9^ While the role of Type II IFNs in epithelial-immune cell signaling in IBD is partly defined,^10^ the involvement of Type I IFNs (IFN-Is) remains poorly understood in the context of epithelial–immune cell interactions. IBD patients who did not respond to anti-TNF therapy showed elevated IFN-I gene expression compared to responders after treatment.^11^^,^^12^ At homeostasis, IFNs condition the epithelium for interaction with the gut microbiome and its metabolic products, supporting the immune signaling secretome (such as the secretion of antimicrobial peptides, defensins, cytokines, and growth factors).^13^^,^^14^ However, persistent and chronic IFN-I signaling during inflammation can be harmful.^9^^,^^14^ Butyrate in a viral inflammatory model of human lung and colonic epithelial cells suppressed the pro-inflammatory secretome by modulating IFN-stimulated gene expression.^15^ However, it remains unclear whether butyrate modulates IFN-I signaling in human IBD, particularly in R versus NR, and whether this modulation affects gut epithelial integrity.

Here, we identify a novel TNF–IFN-I axis in CD that can stratify epithelial responses to therapy. Using patient-derived intestinal organoids, we tested the hypothesis that dysregulated IFN-I signaling contributes to epithelial dysfunction and limits the effectiveness of current therapies. We examined whether modulation of IFN-I signaling and epithelial butyrate sensing could differentially restore epithelial homeostasis across clinically defined anti-TNF CD-Rs and CD-NRs. This study defines IFN-I activity as a feature of epithelial response in CD and provides a rationale for precision, epithelium-targeted treatments.

## 2. Methods

### 2.1. Donor samples and ethical approvals

Crohn’s disease (CD) and healthy non-IBD (controls) donors who provided informed consent were recruited through Mercy and Dunedin Hospitals, Dunedin, New Zealand, with approval from the New Zealand Health and Disability Ethics Committee (Ref# 2023 FULL 15103, 2022 EXP 11704). Sigmoid colon biopsies were collected from macroscopically non-inflamed regions, either from donors with active CD or from non-IBD controls undergoing routine colonoscopy (Table S1). Donor characteristics, clinical response status, treatment exposure, and the distribution of individual donors across experimental figures are summarized in Table S1. CD responders to anti-TNF therapy were defined by treating gastroenterologists as achieving a ≥100-point Crohn’s Disease Activity Index (CDAI) reduction or remission (CDAI < 150) with endoscopic improvement; non-responders did not meet these criteria. During consenting, patients were briefed on the study’s aims, recruitment strategies, and sampling methods, and study findings were communicated to them at annual patient forums. Individual donor samples were used across multiple experimental assays and figures, indicated in Table S1, enabling complementary analyses of donor-specific epithelial responses across experiments.

### 2.2. Human organoid culture, and experiments

Crypts were generated from sigmoid colon biopsies of control (non-IBD) and CD donors using established chelation and digestion methods.^16^^,^^17^^,^^18^ Organoids derived from isolated crypts were cultured in Matrigel® (Corning, NY, USA) with growth factor-enriched medium^16^^,^^17^ and passaged every 8–10 days at a 1:4–6 ratio by mechanical dissociation with a 27 G × ½ needle. Media were changed on alternate days, and experiments were performed from passages 3 to 10. ROCK inhibitor at 10 µM (Merck, Kenilworth, NJ, USA; no. 1293823) was included during amplification but omitted in experimental setups.

Control and CD organoids were seeded at 1 × 10^3^ cells per well (50 µL) in parallel. On day (D)7, they were exposed to 0.1, 1, or 10 mM butyrate in complete medium for 24 h. Butyrate-exposed organoids (D8) were exposed to an inflammatory cytokine cocktail comprising 50 ng/mL TNF (R&D Systems, no. 210-TA-100/CF), 50 ng/mL IL1β (R&D Systems, no. 201-LB-100/CF), and 50 ng/mL IL6 (R&D Systems, no. 7270-IL-100/CF) for an additional 24 h. On D9, butyrate and the inflammatory cytokine cocktail pretreated organoids were exposed to adalimumab (10 µg/mL; kind gift from Dr Paulo C.M. Urbano and Hans J.P.M. Koenen, Radboud University Medical Center, Nijmegen, The Netherlands) or 5 µg/mL adalimumab and 5 µg/mL of the neutralizing antibody to IFN-I (Novus Biologicals, Auckland, New Zealand, no. 21100-1) for 1 h before collection for sample processing.

### 2.3. FITC dextran 4000 uptake

The relative permeability of control and CD organoids exposed to butyrate and/or cytokines and the anti-TNF and/or anti-IFN-I antibody was measured by adding 0.01 mg/mL^16^ fluorescein isothiocyanate (FITC)-labeled dextran, MW 4000 (FD4; Sigma Aldrich, no. 46944) to the growth media on D9 for 24 h at the end of organoid experiments. Median fluorescence intensity (MFI) was determined by measuring the ratio of basolateral to luminal fluorescence using the ImageJ analysis platform.

### 2.4. EdU assay for proliferation and co-localization with CC3 for apoptosis using immunostaining

Whole-mount human organoids (1 × 10³ cells/mL) on eight-well chamber slides were stained using the Click-iT® Plus EdU Alexa Fluor® 488 Imaging Kit (ThermoFisher Scientific; no. C10632) according to the manufacturer’s instructions. For co-localization with cleaved caspase-3 (CC3), EDU-stained organoids were blocked with 1% BSA for 2 h at room temperature (RT), incubated overnight at 4 °C with primary anti-CC3 antibody clone (1:100; Cell Signaling, Danvers, MA, USA; no. 9661), and washed with PBS. Secondary antibody goat anti-rabbit IgG (H + L) clone, Alexa Fluor Plus 488 (1:1000; ThermoFisher Scientific; no. A32731) was added for 1 h, followed by PBS wash and DAPI staining for 10 min. Wells were washed three times with PBS (5 min each) and mounted in ProLong™ Gold Antifade Mountant for imaging on a Zeiss 710 LSM confocal laser scanning system (GmbH, Jena, Germany).

### 2.5. RNA isolation, cDNA synthesis and qRT-PCR

RNA from human organoids was extracted using the Ambion® PureLink® RNA Isolation Kit (ThermoFisher Scientific; no. 12183025). cDNA was synthesized from 50–300 ng RNA per sample with the SuperScript™ III First-Strand Synthesis System (ThermoFisher Scientific; no. 18080051) on a CFX384 Touch™ Real-Time PCR Detection System. Quantitative PCR was performed using TaqMan Fast Advanced Master Mix on an ABI Prism 7700 sequence detection system. Primer/probe oligonucleotides included IL7R* (Hs00902334_m1), IFIH1*(Hs00223420_m1), IFI6* (Hs00242571_m1), IFI44* (Hs00197427_m1), SP110* (Hs00893490_m1), IRF1* (Hs00971965_m1), CXCL10* (Hs00171042_m1), IFIT3* (Hs01922752_s1), and ACTB (Hs01060665_g1). PCR conditions were 95 °C for 3 min, followed by 37 cycles of 94 °C for 15 s, 54 °C for 20 s, and 72 °C for 25 s. Data were analyzed by the ΔΔCt method. IFN-I* target profiling was assessed using the RT^2^ Profiler™ PCR Array Human Interferons & Receptors (GeneGlobe, Germantown, MD, USA; no. PAHS-064ZA-6, 330231) as per the manufacturer’s instructions.

### 2.6. LegendPlex^TM^ profiling and analysis

Cytokine concentrations were measured using the LEGENDplex Human Inflammation Panel 1 (13-plex) with a V-bottom plate multiplex assay (BioLegend; no. 740809). Supernatants from organoid cultures (25 µL, D9) were diluted 1:1 with assay buffer, and 25 µL of diluted sample was used per well. Standard curves were generated by 4-fold serial dilution of known standards across seven points. Diluted samples or standards (25 µL) were added to 96-well plates with 25 µL assay buffer and 25 µL capture beads, followed by 2 h of incubation at RT on a shaker. After washing, 25 µL biotinylated detection antibodies were added for 1 h, then 25 µL streptavidin–phycoerythrin (SA-PE) for 30 min. Plates were washed twice and resuspended in 150 µL wash buffer, and samples were transferred to 5-mL flow cytometry tubes. Data were acquired on a BD LSR Fortessa flow cytometer and analyzed with the LEGENDplex Data Analysis Software Suite (BioLegend, SD, USA), as per the manufacturer’s protocol.

### 2.7. IBD cDNA array and histological grading of parallel tissues

Human IBD cDNA 96-well plates containing cDNA from six healthy controls, 12 UC, and 12 CD donors were obtained from OriGene Technologies (Rockville, MA, USA; no. CCRT501). Histological evaluation by the supplier’s pathologist confirmed 12 active CD, 12 UC, and six non-IBD control samples. Available donor medical histories were provided by OriGene upon request. PCR was performed on an ABI Prism 7700 sequence detection system following the manufacturer’s instructions, using 20× TaqMan primers for IFN-I target genes (as listed above).

### 2.8. Bioinformatics analysis of human IBD gene-expression array databases

Transcript levels of target genes were analyzed using publicly available human IBD datasets from Arijs et al. (2009)^19^ and Smillie et al. (2019)^20^ via the Hegemon platform^21^ from controls and CD donors before and after anti-TNF treatment. Donors were further classified as responders or non-responders to the anti-TNF, infliximab. Raw expression values were imported into GraphPad Prism (v10; GraphPad Software, San Diego, CA, USA) for statistical analysis. Group comparisons were performed using one-way ANOVA with Šidák’s *post hoc* test. Statistical significance was set at *P < .05, **P < .01, ***P < .001, and ****P < .0001.

### 2.9. Murine bulk RNA-sequencing analysis

Murine colonic organoids were generated from TNFR1^−/−^ mice (C57BL/6J background; stock #002818, Jackson Laboratory, ME, USA) and TNFR1^+/−^ littermate controls. All animal experiments were conducted under IACUC protocol #288 (Children’s Hospital Los Angeles, CHLA). Experimental and control groups included male and female mice (8–16 weeks) that were co-housed throughout the study. Prior to tissue collection, animals were anesthetized with isoflurane and killed by cervical dislocation. TNFR1^−/−^ and TNFR1^+/−^-derived organoids (passage 3) were seeded at 1 × 10³ cells/50 µL. On D5, RNA was extracted from the organoids using the Ambion® PureLink® RNA Isolation Kit as per the manufacturer’s protocol. mRNA was prepared for 2 × 75-bp sequencing on a HiSeq 4000 (Illumina). Library preparation and sequencing were performed at the CHLA Single Cell, Sequencing, and CyTOF Core Laboratory (Los Angeles, CA, USA). Reads were mapped to the latest UCSC transcript set with Bowtie2 v2.1.0, and gene expression was estimated using RSEM v1.2.15. Raw counts were normalized with the TMM method, and differentially expressed genes were identified using edgeR (*P* < .05; fold change >1.5). Gene Ontology and pathway enrichment analyses were conducted with ClusterProfiler.

Schematic illustrations for the graphical abstract were drafted using the AI-assisted design tool FigureLabs, followed by manual refinement.

## 3. Results

### 3.1. IFN-I signaling pathway genes are enriched in epithelium of IBD donors and in murine organoids depleted for TNF-receptor 1 signaling

**Figure 1.**
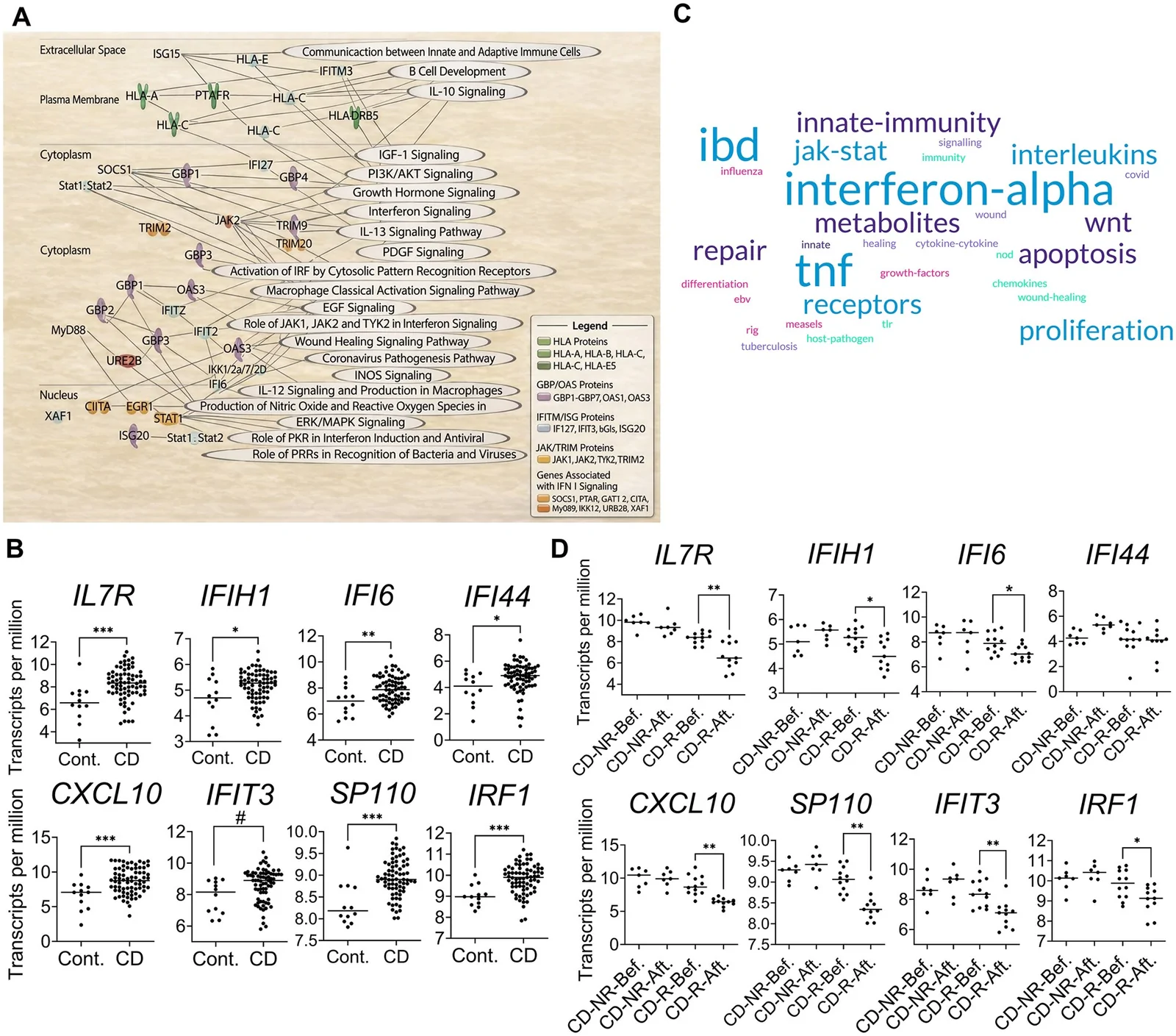
IFN-I target gene networks are altered in human IBD epithelium and TNFR1 ablated intestinal organoids. (A) Ingenuity pathway re-analysis of single cell RNA sequencing data from Smillie et al. (2019),^20^ showing IFN-I target genes and associated pathways enriched in the epithelium of IBD donors. Green: receptors; blue: transcription factors; purple: IFN-inducible genes; orange: ligands/transcription factors. (B) Re-analysis of single cell RNA sequencing data from Arijs et al. (2009),^19^ showing IFN-I target genes specifically induced in the epithelium of CD donors. *N* = 12 non-IBD; *N* = 73 CD. (C) A word cloud determinator showing the IFN-I target pathways downregulated in mouse organoids ablated for the constitutively expressed TNF receptor 1 (TNFR1). Word size reflects the magnitude of downregulation or relative contribution of each gene to the pathway. *N* = 3. Datasets used in these analyses are included in Table S2. (D) Single-cell RNA sequencing data from Arijs et al. (2009),^19^ showing IFN-I target genes in the epithelium of CD donors before and after treatment with the anti-TNF, infliximab. *N* = 12 non-IBDs; *N* = 73 CD; *N* = 7 CD-non-responders (NR); and *N* = 11 CD-responders (R). Data were analysed by an unpaired (B) *t*-test, or one-way ANOVA, with a Sídák post-hoc test (D), where ****P < .0001, ***P < .001, **P < .01, *P < .05. Datasets used in these analyses are included in Table S2.

Previous studies have shown increased IFN-I signaling in IBD NR donors compared with R and healthy controls.^11^^,^^12^ To define the relationship between IFN-I and TNF responses, we performed an integrated analysis of existing human and murine datasets. Re-analysis of a single-cell RNA sequencing dataset^20^ from inflamed intestinal epithelium of IBD donors demonstrated significant enrichment of IFN-I-associated genes (*P* < .0001; enrichment score +0.85; Figure 1A). Ingenuity Pathway Analysis identified upregulation of growth factor signaling, pattern recognition receptor pathways, innate immune activation, antiviral responses, metabolic programs, and epithelial restitution pathways (Figure 1A). To determine whether IFN-Is were enriched in the intestinal mucosa of IBD patients, particularly those with CD,^19^ we reanalyzed a publicly available RNA-Seq dataset.^19^ In this analysis, multiple IFN-I-associated genes were significantly upregulated in CD donors relative to non-IBD donors, including *IL7R* (*P* = .0003), *IFIH1* (*P* = .017), *SP110* (*P* = .002), *IFI44* (*P* = .040), *CXCL10* (*P* = .0004), *IFI6* (*P* = .0079), and *IRF1* (*P* = .0006) (Figure 1B). IFN-I target genes were upregulated in the intestinal mucosa of CD patients, suggesting enhanced IFN-I signaling as a feature of disease-associated epithelial responses.

To mechanistically investigate whether epithelial TNF signaling influences IFN-I-associated transcriptional programs, we generated organoids from TNFR1-deficient (TNFR1^−/−^) mice, profiled at baseline (unstimulated conditions), and performed bulk RNA Seq (Figure 1C; Figure S1A-C). We used TNFR1-deficient organoids as a reductionist mechanistic model to specifically investigate whether epithelial TNF signaling is required to regulate IFN-I-associated transcriptional programs. This approach allowed us to isolate epithelial-intrinsic TNF/TNFR1 signaling from confounding immune and stromal influences present *in vivo*. Given the known cross-talk between TNF and IFN pathways, we sought to determine whether disruption of TNF signaling directly alters epithelial IFN-I responses. Loss of TNFR1 led to significant downregulation of IFN-I-responsive genes (*P* < .001; enrichment score −0.68; Figure S1) and reduced IFN-I stimulated networks (Figure 1C) controlling innate-immunity, TNF-signaling, proliferation, metabolism, antiviral-defense, apoptosis, antigen-presentation, and epithelial repair compared with TNFR1^+/−^ controls. These results indicate that TNFR1 loss diminishes IFN-I-driven gene programs essential for host defense and epithelial homeostasis.

To determine whether these TNF-dependent IFN-I responses observed in murine epithelium were reflected in human IBD, we examined IFN-I target gene expression in intestinal mucosa from CD patients undergoing anti-TNF therapy. We re-analyzed the same single RNA-Seq dataset^22^ shown in Figure 1B. In responders (R-CD), anti-TNF treatment led to significant reductions in transcript levels of *IL7R* (*P* = .0073), *IFIH1* (*P* = .030), *SP110* (*P* = .0026), *CXCL10* (*P* = .0013), *IFIT3* (*P* = .0059), *IFI6* (*P* = .014), and *IRF1* (*P* = .029; Figure 1D); no significant changes were observed in non-responders (NR-CD). These data suggest that sustained IFN-I gene expression in NR-CD donors may be associated with clinical non-responsiveness. To validate these results, we performed a cDNA assay on intestinal mucosa from an IBD cohort (*n* = 3 NR-CD, *n* = 7 R-CD on anti-TNF therapy, and *n* = 11 CD donors without clinical data; Figure S1D). IFN-I target genes were elevated in NR-CD donors versus non-IBD donors, while R-CD donors showed no increases.

To confirm that IFN-I upregulation was linked to TNF rather than general inflammation or other therapies, we examined IFN-I target gene expression in CD patients receiving ustekinumab^22^ (Figure S2A), azathioprine^23^ (Figure S2B), or vedolizumab^24^ (Figure S2C). IFN-I genes were elevated in CD donors, but no consistent differences between NR- and R-CDs were observed, except for lower *IRF1*, *CXCL10*, and *IFIT3* in R-CDs on azathioprine, highlighting that the strong IFN-I signature in CD mucosa is most closely associated with TNF-driven inflammation. These findings demonstrate that TNF signaling modulates epithelial IFN-I responses and that TNF–IFN-I crosstalk coordinately shapes IFN-I gene expression during inflammation.

### 3.2. Butyrate differentially regulates epithelial proliferation and IFN-I expression in CD donors

**Figure 2.**
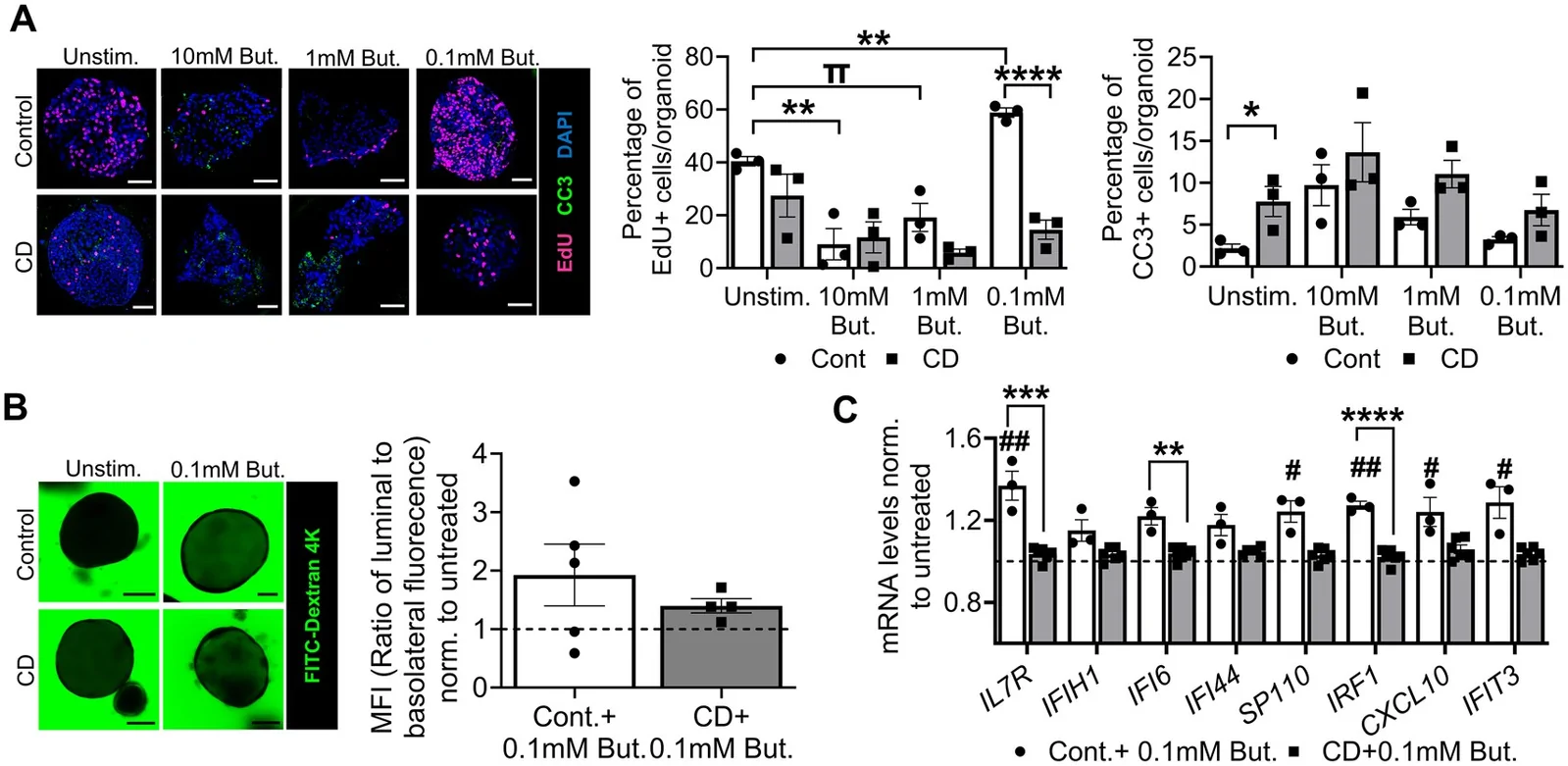
Butyrate regulates epithelial proliferation, permeability, and IFN-I responses in non-IBD and Crohn’s disease organoids. The effect of increasing concentrations of butyrate (0.1, 1, and 10 mM) (72 h) exposure on (A) the percentage of EdU, and CC3^+^ cells per organoid (*N* = 3); (B) permeability of the epithelium determined by translocation of FITC-Dextran 4K across the epithelium (*N* = 4–6); and (C) mRNA levels of IFN-I targets in organoids derived from non-IBD (non-IBD) and CD donors when exposed to 0.1 mM butyrate (*N* = 3–5). Representative images are depicted in A and B. Scale bars = 100 µm. Data were analyzed by a one-way ANOVA with a Sídák post-hoc test, where, # represents values normalized to untreated non-IBD: #*P* < .05, ##*P* < .01, ###*P* < .001, ^π^*P* = 0.05–0.09 and asterisks represent comparisons between groups: *P < .05, **P < .01, ***P < .001, ****P < .0001. Dotted line indicates the non-IBD at 1. Donors included in these analyses are shown in Table S1 and donor-derived organoid lines included specifically in this analysis are listed in Table S3.

Given the association between TNF-dependent IFN-I signaling and epithelial metabolic pathways, we investigated the effects of the bacterial metabolite butyrate on epithelial barrier-function. As luminal butyrate is reduced in IBD,^8^ we examined whether treatment with 0.1, 1, or 10 mM differentially affected epithelial integrity in CD versus non-IBD organoids. Barrier integrity was assessed by measuring epithelial-turnover, proliferation and apoptosis, and permeability using an FITC dextran 4K translocation assay.^16^ The 0.1 mM dose reflects depleted luminal levels in IBD;^25^ 10 mM corresponds to physiological concentrations in the healthy colon.^25^ CD organoids exhibited a higher frequency of CC3^+^ apoptotic cells compared with organoids from non-IBD donors (*P* = .04; Figure 2A), while no significant differences were observed in 5-ethynyl-2′-deoxyuridine (EdU)^+^ proliferating cells (Figure 2A). In non-IBD organoids, higher butyrate concentrations (1 and 10 mM) reduced EdU^+^ cell levels, whereas 0.1 mM butyrate increased proliferation (*P* = .0018; Figure 2A). Butyrate did not significantly alter proliferation in CD organoids at any concentration. CC3^+^ apoptotic cell levels remained unchanged in both non-IBD and CD organoids (Figure 2A). In summary, low-dose butyrate enhanced proliferation only in non-IBD organoids, whereas CD organoids showed no proliferative or apoptotic response at any dose.

We next evaluated the impact of 0.1 mM butyrate on epithelial barrier-function. This dose did not alter FITC dextran 4K uptake in CD or non IBD organoids (Figure 2B); however, higher concentrations (1–10 mM) increased permeability and reduced stem cell expression in both groups (Figure S3A, B). CD organoids showed lower stem cell markers (Figure S3B) and 80% reduction in *Lgr5* mRNA after 10 mM versus 0.1 mM butyrate, indicating loss of repair competent stem cells (Figure S3C). Baseline (no butyrate) MUC2 mRNA expression was comparable between control (non-IBD) and CD-derived organoids. Butyrate stimulation (10 or 0.1 mM) selectively enhanced MUC2 expression in CD-derived organoids relative to controls (Figure S3B, C). Hence, 0.1 mM butyrate was selected as the optimal dose as it maintained epithelial integrity and stem cell-associated repair capacity while inducing MUC2 expression in CD organoids.

We next assessed the effects of low-dose butyrate on IFN-I gene expression in non-IBD and CD organoids (Figure 2C), based on Figure 1 and studies that showed butyrate can selectively regulate IFN-I gene expression and modulate the immune secretome in colonic epithelial cells.^18^ We analyzed >80 IFN-I pathway genes (data not shown) in non-IBD and CD organoids (Table S1) in response to butyrate and found that *IL7R*, *IFI6*, *IFI44*, *IFIH1*, *SP110*, *CXCL10*, *IRF1*, and *IFIT3* were specifically elevated in CD.^9^^,^^26^^,^^27^^,^^28^^,^^29^^,^^30^^,^^31^^,^^32^ Exposure of non-IBD organoids to 0.1 mM butyrate significantly increased the expression of *IL7R*, *SP110*, *IRF1*, and *IFIT3* relative to untreated levels (Figure 2C). Addition of butyrate did not alter the expression of IFN-I target genes in CD organoids (Figure 2C). CD organoids exposed to 0.1 mM butyrate had significantly lower expression of *IL7R* (24.2%, *P* = .0003), *IFI6* (14.6%, *P* = .001), and *IRF1* (19.6%, *P* < .0001) compared to non-IBD organoids treated with 0.1 mM butyrate (Figure 2C). These findings indicate that epithelial responses to butyrate are impaired in CD.

### 3.3. Butyrate restores inflammation-induced increase in epithelial integrity and IFN-I expression in anti-TNF-responsive but not non-responsive CD organoids

**Figure 3.**
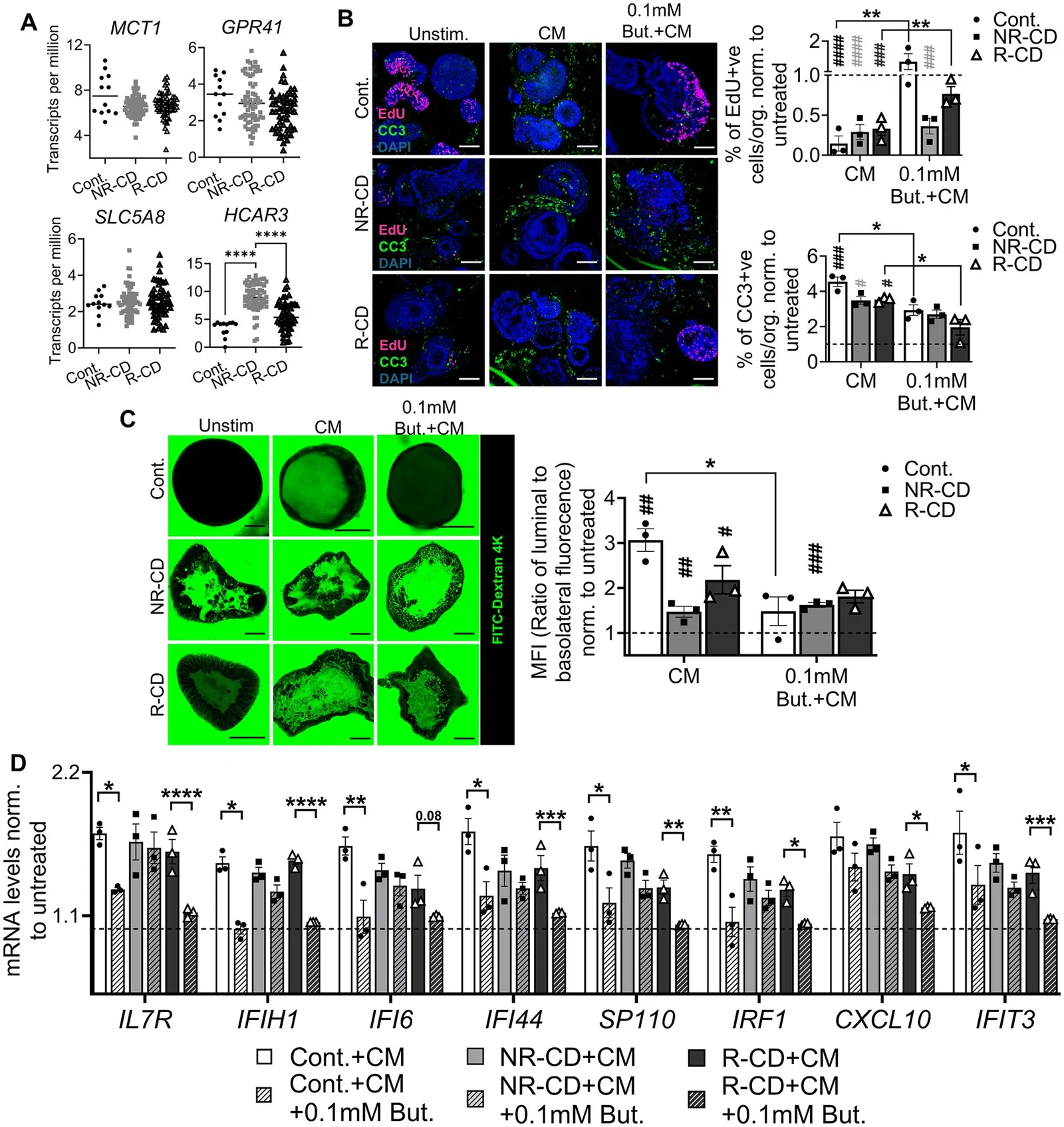
Butyrate rescues inflammation-dependent disruption of epithelial integrity and IFN-I expression in R-CD and non-IBD organoids, but not in NR-CD epithelia. (A) Re-analysis of single cell RNA sequencing data from Arijs et al. (2009),^19^ showing butyrate receptor genes specifically in the intestinal mucosa of CD donors responsive or non-responsive to the anti-TNF antibody, infliximab. *N* = 12, non-IBDs; *N* = 66, NR-CD; and *N* = 55, R-CD. Non-responders (NR), responders (R). Data were analyzed by a one-way ANOVA with a Sídák post-hoc test, where # represents values normalized to untreated non-IBD. ***P < .001, ****P < .0001. (B) The effect of cytokine mix (CM) (48 h; 50 ng/mL TNF, IL1β and IL6) ± 0.1 mM butyrate (72 h) exposure on the percentage of EdU, and CC3^+^ cells per organoid in organoids derived from non-IBD (non-IBD) and CD donors (R, responder; NR, non-responder). Representative images show the localization of EdU^+^ (pink) and CC3^+^ (green) cells in nuclear-DAPI (blue) immunofluorescent organoids. Scale bars = 200 µm. (C) The effect of CM ± 0.1 mM butyrate (72 h) exposure on the permeability of the epithelium as determined by translocation of FITC dextran across the epithelium in organoids derived from non-IBD and NR- and R-CD donors. Representative images show the fluorescent FITC-Dextran 4K in the basal and luminal compartment of the organoids. Scale bars = 200 µm. (D) The effect of CM on the mRNA levels of IFN-I targets in organoids derived from non-IBD and NR- and R-CD donors when exposed to 0.1 mM butyrate (72 h). *N* = 3. Data were analyzed by a one-way ANOVA with a Sídák post-hoc test, where *P < .05, *P < .01, **P < .001, ****P < .0001. Dotted line indicates non-IBD at 1. Donors included in these analyses are shown in Table S1 and donor-derived organoid lines included specifically in this analysis are listed in Table S3. The dataset used in this analysis is included in Table S2.

We then investigated whether addition of butyrate could restore epithelial function in inflammatory conditions seen in CD. Butyrate was selected not only because it is reduced in CD, but also because it plays a key role in epithelial metabolism, barrier repair, and immune regulation. In our bulk RNA-seq analysis of TNFR1-deficient murine organoids, loss of epithelial TNFR1 signaling suppressed IFN-I-responsive genes and altered metabolic pathways, supporting a close relationship between epithelial IFN-I signaling and metabolic homeostasis. Consistent with this, emerging evidence suggests a link between butyrate, IFN-I-associated pathways, and downstream innate immune signaling, potentially mediated through metabolic and epigenetic mechanisms. We therefore hypothesized that butyrate may restore epithelial function by modulating IFN-I-associated inflammatory and metabolic programs. We examined the expression of butyrate receptors in intestinal mucosa from non-IBD and CD donors, including those clinically responsive (R-CD) or non-responsive (NR-CD) to the anti-TNF antibody infliximab (Figure 1D).^19^ Our results showed that butyrate uptake receptors,^33^^,^^34^  *MCT1*, *GPR41*, and *SLC5A8*, were expressed at similar levels in non-IBD, NR- and R-CD donors (Figure 3A). However, expression of the butyrate receptor *HCAR2*^33^ was not detected in any CD group. *HCAR3*,^34^ which shares 99% homology with *HCAR2*, was higher in NR-CD donors compared to non-IBD donors, and to R-CD donors (Figure 3A). R-CD donors had no significant difference in the levels of the butyrate receptors compared to non-IBD donors. Consistently, transcript levels of *MCT1*, *GPR41*, and *GPR43* were comparable between organoids derived from control (non-IBD) and R-CD or NR-CD patients (Figure S4A). The levels of *SLC5A8* were undetectable in organoids at baseline (no butyrate). Unlike the native intestinal mucosa of patients (Figure 3A), however, organoids derived from NR-CD patients showed comparable levels of *HCAR3* to organoids derived from control and R-CD patients (Figure S4A).

To mimic the complex intestinal environment in IBD, we established an *in vitro* inflammatory colonic organoid model using biopsies from non-IBD and CD donors classified as anti-TNF responders (R-CD) or non-responders (NR-CD) to adalimumab (R: ≥100-point CDAI reduction or remission [CDAI < 150] with endoscopic improvement; NR: no response). Patients were stratified according to anti-TNF response as responders, primary non-responders, and secondary non-responders, the last including those who developed anti-TNF antibodies. Patients who discontinued anti-TNF therapy due to adverse events or other non-efficacy-related clinical reasons were excluded from response classification. The NR-CD cohort included primary and secondary non-responders, with variable anti-TNF exposure at sampling, including low or undetectable drug levels and ongoing treatment. Organoids were exposed to a pro-inflammatory cytokine cocktail (CM: 50 ng/mL TNF, IL1β, IL6)^13^ to simulate CD-like inflammation and epithelial damage and used to assess butyrate effects on epithelial-turnover and barrier-function across donor groups. To characterize the effects of the CM on epithelial function, we first examined the contribution of individual cytokines to epithelial injury and barrier dysfunction. TNF, IL1β, and IL6 each significantly suppressed epithelial proliferation (EdU^+^) and increased apoptosis (CC3^+^), which was associated with increased epithelial permeability in both control and CD organoids relative to untreated controls (Figure S4E, F). CM exposure reduced EdU^+^ proliferation and increased CC3^+^ apoptosis in all groups (Figure 3B). Addition of 0.1 mM butyrate restored proliferation in CM-treated non-IBD organoids and increased EdU^+^ cells in R-CD organoids but had no effect in NR-CD organoids. CC3^+^ apoptosis decreased with butyrate in non-IBD and R-CD organoids, while NR-CD organoids were unchanged. Baseline permeability was higher in R-CD and NR-CD versus non-IBD organoids (Figure 3C); CM increased permeability 3-fold in non-IBD and 1.4- and 2.1-fold in NR-CD and R-CD organoids, respectively. Butyrate reduced CM-induced permeability in non-IBD organoids but had no effect in R-CD and NR-CD organoids. These data show that 0.1 mM butyrate promotes epithelial repair in non-IBD and R-CD organoids by restoring proliferation and limiting apoptosis but does not improve turnover or barrier-function in NR-CD organoids.

Increased permeability correlated with morphological abnormalities in CD organoids, including apical erosions, epithelial fragility, and multi-luminal structures (Figure 3C; Figure S4A). NR-CD mucosal tissue displayed crypt distortion, atrophy, crypt loss, surface erosions or ulceration, and active inflammation with cryptitis and crypt abscesses (Figure S4B). Overall epithelial architecture was disrupted, with more severe damage in NR-CDs.

These findings raise the question of whether the effect of butyrate on proliferation and apoptosis is mediated via IFN-I signaling and if it can promote epithelial repair through this pathway. CM treatment increased transcript levels of *IL7R*, *IFIH1*, *IFI6*, *IFI44*, *SP110*, *IRF1*, *CXCL10*, and *IFIT3* (>1.5-fold; *P* < .05) in non-IBD, R-CD, and NR-CD organoids (Figure 3D and Figure S4C). Addition of butyrate significantly reduced CM-induced expression of IFN-I target genes in non-IBD and R-CD organoids but had no effect on NR-CD organoids (Figure 3D). These findings indicate that butyrate-associated restoration of epithelial proliferation and reduction of apoptosis occurs with suppression of inflammation induced IFN-I target gene expression. This repair response is present in non-IBD and R-CD organoids but absent in NR-CD organoids, consistent with reduced epithelial responsiveness to butyrate during inflammation.

### 3.4. Differential effects of anti-IFN-I antibody treatment and butyrate supplementation on epithelial integrity and IFN-I expression

**Figure 4.**
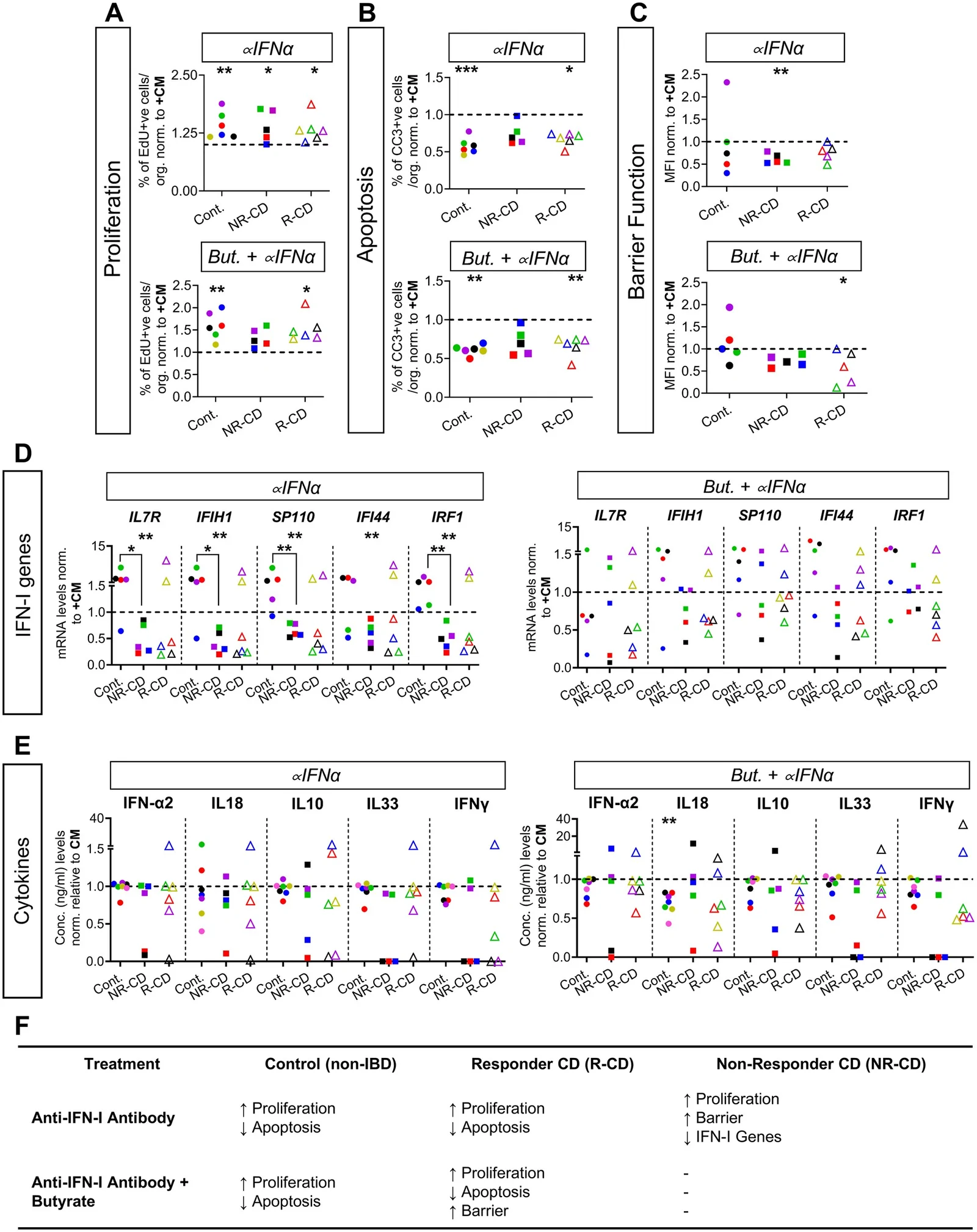
Butyrate and anti-IFN-I antibody treatment rescues loss in epithelial integrity in a donor-specific manner for CD donors. The effect of 5 µg/mL anti-IFN-I antibody treatment (αIFNα) on cytokine mix (CM) (48 h; 50 ng/mL TNF, IL1β, and IL6) ± 0.1 mM butyrate (72 h) was assessed on: (A) the percentage of EdU^+^ cells per organoid; (B) the percentage of CC3^+^ cells per organoid; (C) epithelial permeability, determined by FITC-dextran translocation across the epithelium in organoids; (D) mRNA levels of IFN-I target genes; and (E) cytokine levels in supernatants quantified by cytokine bead array (LEGENDplex™) in organoids derived from non-IBD (non-IBD) and CD donors (R, responder; NR, non-responder), measured by qPCR. *N* = 5–7. Data were analyzed by one-way ANOVA with Kruskal-Wallis test, where *P < .05, **P < .01, ***P < .001, ****P < .0001. Dotted line indicates values normalized to CM at 1. (F) Summary of the effects of butyrate and anti-IFN-I antibody on epithelial integrity, cytokines, and IFN-I gene expression in organoids derived from control (non-IBD) and CD donors. Donors included in these analyses are shown in Table S1 and donor-derived organoid lines included specifically in this analysis are listed in Table S3.

To assess whether modulating butyrate availability and IFN-I signaling provides therapeutic benefit in CD, we tested butyrate supplementation and IFN-I blockade in intestinal organoids. Consistent with known heterogeneity in epithelial and immune responses to IBD-associated inflammation,^16^^,^^17^^,^^18^^,^^35^ donor-specific responses were observed. Despite this variability, anti-IFN-I antibody treatment consistently increased epithelial proliferation in CM-treated non-IBD (*P* = .003), NR-CD (*P* = .016), and R-CD (*P* = .018) organoids, restoring proliferation to approximately 38% (Figure 4A and Figure S5A). Combined anti-IFN-I antibody and low-dose butyrate (0.1 mM) further increased proliferation to ∼55% in non-IBD (*P* = .005) and R-CD (*P* = .028) organoids compared with CM alone but had no effect in NR-CD organoids (Figure 4A and Figure S5A). Anti-IFN-I antibody treatment alone or with butyrate significantly reduced epithelial apoptosis in non-IBD (*P* = .009) and R-CD (*P* = .041) organoids, with no change observed in NR-CD organoids (Figure 4B and Figure S5A). Anti-IFN-I antibody treatment alone reduced CM-induced permeability in NR-CD organoids (*P* = .044), with no significant effect in non-IBD or R-CD organoids (Figure 4C and Figure S5B). In contrast, combining butyrate with anti-IFN-I antibody decreased permeability in R-CD organoids, without affecting NR-CD or non-IBD organoids (Figure 4C and Figure S5B).

We next investigated whether anti-IFN-I antibody, alone or with butyrate, modulated IFN-I gene expression. Anti-IFN-I antibody treatment significantly decreased transcript levels of *IL7R*, *IFIHI*, *SP110*, *IFI44*, and *IRF1* in NR-CD organoids, but had no effect in non-IBD or R-CD organoids (Figure 4D and Figure S5C). Combining anti-IFN-I antibody with butyrate did not further alter CM-induced IFN-I gene expression. These findings indicate that anti-IFN-I antibody suppressed cytokine-induced IFN-I gene expression and improved proliferation in NR-CD organoids, while butyrate alone restored epithelial integrity in R-CD organoids without altering IFN-I gene expression.

Given that cytokine expression in IBD has been associated with barrier defects,^46^ we next investigated whether anti-IFN-I antibody modulated cytokine expression. Analysis of CM-induced epithelial cytokines in non-IBD, NR-CD, and R-CD organoids revealed no significant changes in response to either treatment (Figure 4E and Figure S5D). Anti-IFN-I antibody and butyrate had distinct effects across donor groups, showing that responses in CM-treated non-IBD organoids do not predict outcomes in Rs or NRs (Figure 4F). Anti-IFN-I antibody restored proliferation and reduced apoptosis in non-IBD and R-CD organoids, with butyrate further enhancing barrier integrity in R-CD organoids, while in NR-CD organoids, it selectively induced proliferation, suppressed IFN-I genes, and improved barrier-function.

### 3.5. Combinational therapy of anti-IFN-I antibody, butyrate, and anti-TNF exhibit donor-specific effects in CD

We assessed clinically relevant combinations: butyrate with anti-TNF, and butyrate with both anti-TNF and anti-IFN-I antibody treatment. These experiments allowed us to evaluate how these combination therapies could modulate epithelial integrity in organoids from patients classified as R-CD or NR-CD (based on clinical and endoscopic criteria; Table S1) to anti-TNF therapy and to explore potential donor-specific therapeutic strategies.

**Figure 5.**
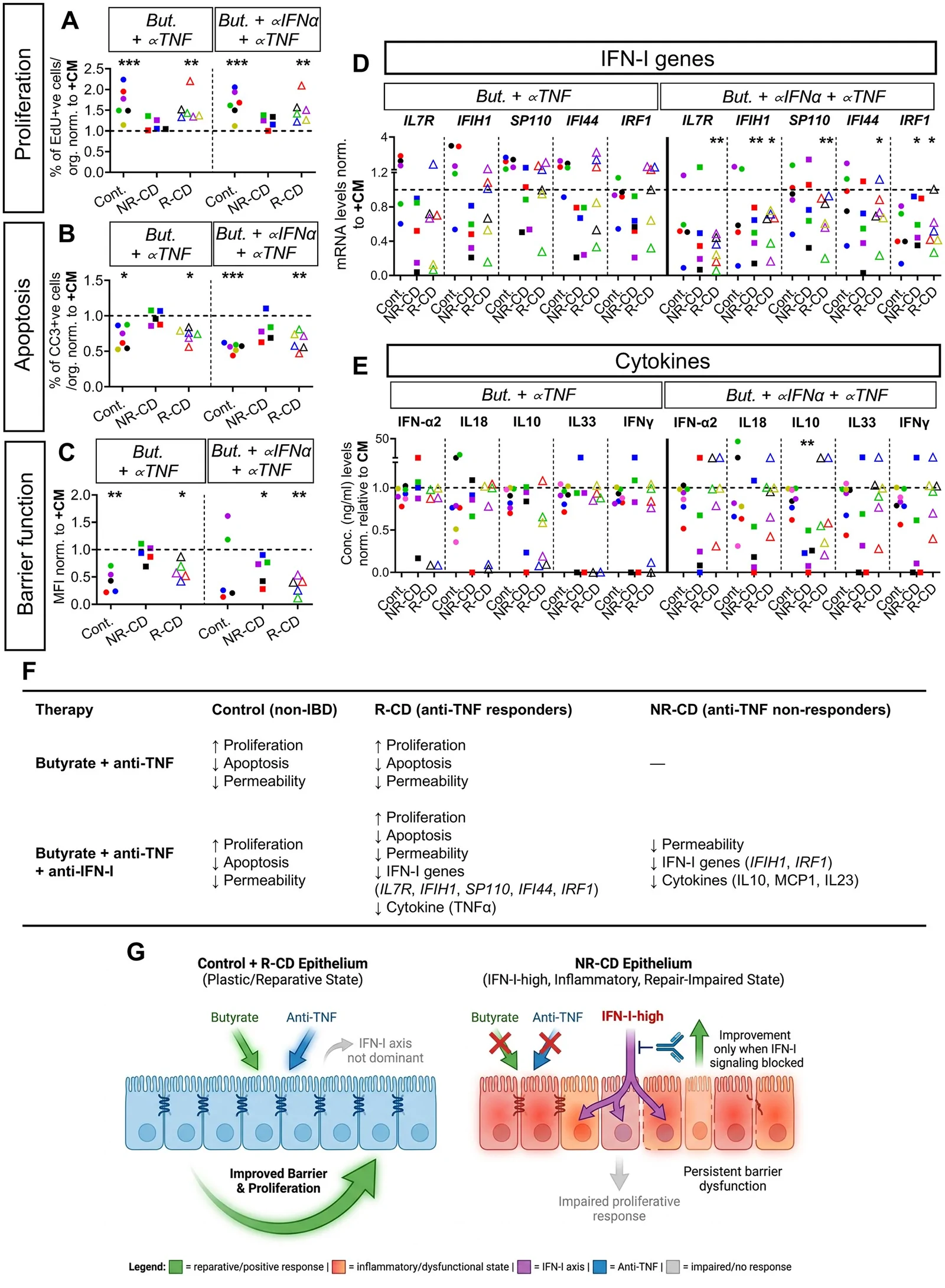
Butyrate and butyrate in combination with anti-TNF and anti-IFN-I antibody treatment rescues loss in epithelial integrity in a donor-specific manner for CD donors treated with anti-TNFs. The effect of 0.1 mM butyrate (72 h) ± 5 µg/mL anti-TNF antibody (αTNF) (infliximab) or 0.1 mM butyrate (72 h) ± 5 µg/mL anti-TNF antibody and 5 µg/mL anti-IFN-I antibody treatment on cytokine mix (CM) (48 h; 50 ng/mL TNF, IL1β, and IL6) was assessed on: (A) the percentage of EdU^+^ cells per organoid; (B) the percentage of CC3^+^ cells per organoid; (C) epithelial permeability, determined by FITC-dextran translocation across the epithelium in organoids; (D) mRNA levels of IFN-I target genes; and (E) cytokine levels in supernatants quantified by cytokine bead array (LEGENDplex™) in organoids derived from non-IBD (non-IBD) and CD donors (R, responder; NR, non-responder), measured by qPCR. *N* = 5–7. Data were analyzed by one-way ANOVA with Kruskal-Wallis test, where *P < .05, **P < .01, ***P < .001, ****P < .0001. Dotted line indicates values normalized to CM at 1. (F) Summary of the effects of butyrate and butyrate in combination with anti-TNF and anti-IFN-I antibody on epithelial integrity, cytokines, and IFN-I gene expression in organoids derived from control (non-IBD) and CD donors. (G) Schematic summarizing the differential epithelial states and responses in colonic organoids derived from responder (R-CD) and non-responder (NR-CD) patients. Donors included in these analyses are shown in Table S1 and donor-derived organoid lines included specifically in this analysis are listed in Table S3.

Butyrate combined with anti-TNF antibody, and the combination of butyrate, anti-TNF, and anti-IFN-I antibody, significantly increased epithelial proliferation in non-IBD and R-CD organoids, but not in NR-CD organoids, relative to CM-treated levels (Figure 5A and Figure S6A). Both treatment combinations also significantly reduced apoptosis in non-IBD and R-CD organoids, but not NR-CD organoids (Figure 5B and Figure S6A). Butyrate with anti-TNF antibody reduced CM-induced permeability in non-IBD and R-CD organoids, whereas the triple combination decreased permeability in NR-CD and R-CD, with no significant effect in controls (Figure 5C and Figure S6B). Combined butyrate and IFN-I blockade with anti-TNF improved epithelial-turnover and barrier-function in non-IBD and R-CD organoids, while in NR-CD organoids only partial barrier restoration was observed.

Next, we assessed the effects of butyrate with anti-TNF antibody and the triple combination of butyrate, anti-TNF, and anti-IFN-I antibody on IFN-I gene expression in non-IBD, NR-CD, and R-CD organoids. The triple combination, but not butyrate + anti-TNF, significantly reduced transcript levels of *IFIH1* and *IRF1* specifically in NR-CD organoids, and *IL7R*, *IFIH1*, *SP110*, *IFI44*, and *IRF1* in R-CD organoids, relative to cytokine-treated controls, with no effect observed in non-IBD organoids (Figure 5D and Figure S6C). Consistent with its lack of effect on IFN-I gene expression, butyrate plus anti-TNF did not alter CM-induced epithelial expression in any donor group (Figure 5E, Figure S6D). In contrast, the triple combination selectively reduced CM-induced IL10, MCP1, and IL23 in NR-CD organoids and TNF in R-CD organoids, with no changes in non-IBD organoids (Figure 5E, Figure S6D). The triple combination restored epithelial homeostasis in NR-CD organoids by reducing permeability, *IFIH1*/*IRF1*, and IL10, MCP1, and IL23 (Figure 5F). In R-CD organoids, it improved barrier-function alongside reduced IFN-I gene expression and TNF production. Barrier integrity in non-IBD organoids improved with butyrate plus anti-TNF and the triple combination (Figure 5F). We interpret these data to indicate that NR-CD organoids are characterized by an IFN-I-high epithelial state that is associated with impaired epithelial repair, barrier dysfunction, and reduced responsiveness to butyrate and anti-TNF therapy compared with R-CD organoids (Figure 5G). Donor-level analyses reveal individual variability in restoring epithelial integrity, IFN-I signaling, and cytokine balance, highlighting the need for precision-medicine approaches.

## Discussion

Despite extensive use of anti-TNF therapy in CD, current treatments fail to achieve clinical benefit in a substantial proportion of patients, and the epithelial mechanisms underpinning therapeutic non-responsiveness remain poorly defined. While IFN-I signaling has been repeatedly linked to anti-TNF failure,^11^^,^^12^^,^^35^ its epithelial specific role and interaction with metabolites such as butyrate have not been resolved. Here, we identify a TNF-dependent IFN-I transcriptional program as a central regulator of epithelial homeostasis in CD. We show that IFN-I target genes are enriched in inflamed CD epithelium and persist in anti-TNF non-responders, that epithelial IFN-I signaling requires intact TNFR1 signaling *in vivo*, and that epithelial responses to butyrate are fundamentally altered in CD. Critically, we demonstrate that restoration of damaged epithelial proliferation, apoptosis, and barrier integrity is donor-specific and depends on coordinated modulation of TNF and IFN-I pathways, with butyrate primarily benefiting anti-TNF responders and IFN-I blockade selectively restoring epithelial function in non-responders. Together, these findings identify epithelial TNF–IFN-I crosstalk as a feature associated with therapeutic response and support stratified, epithelium-targeted combination strategies in CD.

IFN-I pathway genes were enriched in CD donors, with persistent IFN-I-associated signaling most evident in anti-TNF non-responders, while responders showed lower IFN-I-associated gene expression. This observation is consistent with prior studies showing functional crosstalk between TNF and IFN-I signaling in several immune-mediated inflammatory diseases (IMIDs), including IBD,^11^ rheumatoid arthritis,^12^ lupus,^36^ and psoriasis.^37^

To further investigate this relationship, we used TNFR1-deficient murine organoids as a mechanistic model to examine whether epithelial-intrinsic TNF signaling regulates IFN-I-associated transcriptional programs. While this model does not fully recapitulate the complex epithelial and immune landscape observed in anti-TNF non-responsive CD patients, it provides mechanistic evidence that disruption of TNFR1 signaling alters epithelial IFN-I pathway gene expression. Together, these findings support a potential epithelial TNF–IFN-I axis contributing to the IFN-I-high epithelial state observed in anti-TNF-treated CD donors.

CD treatments enhanced epithelial repair in a donor-specific, anti-TNF-dependent manner, highlighting the limitations of using non-IBD donors to model disease-specific inflammation. Each donor displayed a unique profile of epithelial-turnover, barrier-function, and cytokine responses, resulting in variable responses to the treatments. These findings emphasize the importance of personalized therapeutic strategies.

Importantly, our findings indicate that epithelial responses to butyrate in CD are not driven by differences in uptake or receptor expression but instead reflect intrinsic differences in epithelial repair capacity. Comparable expression of canonical butyrate transporters in bulk tissue and unchanged receptor expression in organoids suggest that ligand sensing is preserved across groups. However, data from organoids demonstrate a clear stratification in functional responsiveness, with responder-derived epithelium retaining butyrate-induced proliferative and barrier repair capacity, whereas non-responder epithelium remained refractory. This supports a model in which epithelial state and downstream repair programs, rather than receptor abundance, determine metabolic responsiveness.

NR-CD organoids exhibited reduced responsiveness to butyrate, suggesting an intrinsic butyrate-resistant phenotype. Although HCAR3 gene expression was elevated in NR-CD mucosal tissue, this was not retained in organoid cultures, indicating that HCAR3 upregulation may depend on inflammatory signals from the *in vivo* mucosal microenvironment. Given that butyrate acts through multiple receptor-dependent and receptor-independent pathways, reduced butyrate responsiveness is unlikely to be explained by receptor expression alone. Instead, we propose that the IFN-I-high epithelial state in NR-CD may promote metabolic dysfunction and reduced epithelial plasticity, limiting repair responses to butyrate.

Restoration of epithelial proliferation by butyrate in non-IBD and anti-TNF responsive CD organoids occurred in parallel with suppression of cytokine-induced IFN-I target gene expression, supporting a close association between epithelial repair states and modulation of IFN-I signaling.^38^^,^^39^^,^^40^ In contrast, IFN-I gene expression remained elevated in NR-CD organoids despite butyrate exposure. IFN-I signaling in the intestinal epithelium may reflect sustained epithelial stress or damage in chronic inflammation,^40^^,^^41^^,^^42^ rather than functioning exclusively as a protective response.^40^ While our data do not establish causality, they identify a coordinated epithelial program in which effective metabolic support aligns with dampening of IFN-I responses,^15^^,^^43^ a relationship that is preserved in R-CDs but lost in NR-CDs.

The differential impact of IFN-I blockade in NR-CD, but not non-IBD or R-CD organoids, suggests that epithelial IFN-I-associated programs represent a disease-linked epithelial state maintained independently of TNF-driven inflammation.^44^^,^^45^^,^^46^^,^^47^ Our data support a model in which R-CD and NR-CD organoids represent distinct epithelial states. R-CD organoids retain a relatively plastic, reparative epithelial phenotype that remains responsive to butyrate and anti-TNF therapy, as evidenced by improved proliferation, reduced apoptosis, and enhanced barrier integrity, suggesting that epithelial dysfunction in responders remains largely reversible and responsive to TNF blockade.

In contrast, NR-CD organoids exhibit a distinct IFN-I-high epithelial state characterized by impaired repair, persistent barrier dysfunction, and reduced responsiveness to butyrate plus anti-TNF treatment alone. The restoration of barrier function and suppression of IFN-I-associated genes following anti-IFN-I treatment, together with the limited efficacy of butyrate and anti-TNF alone, suggest that epithelial IFN-I signaling is an important pathway sustaining the anti-TNF non-responder state. We propose that elevated IFN-I signaling within a broader inflammatory network shifts the NR-CD epithelium toward a chronic inflammatory, metabolically altered and barrier-disrupted state that constrains epithelial plasticity, restitution, and sensitivity to TNF blockade. However, our findings indicate that epithelial dysfunction and repair are multidimensional and cannot be attributed to IFN-I signaling alone. Butyrate plus anti-TNF improved proliferation and reduced apoptosis, consistent with metabolic and reparative effects, without requiring direct suppression of IFN-I-associated gene expression. Conversely, the triple combination of butyrate, anti-IFN-I, and anti-TNF improved epithelial permeability despite minimal effects on proliferation or apoptosis, indicating that barrier restoration may be regulated through mechanisms distinct from those governing cellular repair. Thus, IFN-I signaling may contribute to epithelial dysfunction by constraining epithelial plasticity and repair capacity, while additional pathways independently regulate specific functional outcomes, including proliferation, apoptosis, and barrier integrity. These findings support a precision framework in which epithelial-targeted combination therapies are selected according to patient-specific epithelial states and the distinct mechanisms underlying epithelial dysfunction.

JAK inhibition may partially recapitulate the effects of anti-IFN-I therapy by suppressing downstream IFN-I signaling.^48–50^ For example, JAK inhibitors such as tofacitinib, upadacitinib, and filgotinib suppress JAK-STAT signaling downstream of IFN-Is, but also broadly inhibit signaling from multiple cytokines including IL-6, IL-12, IL-23, and γ-chain cytokines.^48^^,^^49^ In contrast, selective anti-IFN-I blockade specifically targets IFN-I signaling pathway markers without directly affecting these parallel inflammatory pathways.^51^^,^^52^ Therefore, while both approaches may improve epithelial repair in IFN-I-high disease states, JAK inhibition likely exerts broader immunomodulatory effects beyond IFN-I suppression.

Overall, our findings suggest that responder and non-responder CD epithelia exist in fundamentally distinct functional states. R-CD organoids retain a metabolically responsive and reparative epithelial phenotype, demonstrating preserved sensitivity to butyrate and anti-TNF-mediated restoration of proliferation, survival, and barrier function. In contrast, NR-CD organoids display a distinct IFN-I-high epithelial state characterized by persistent barrier dysfunction and impaired repair capacity. This state appears relatively resistant to butyrate and anti-TNF therapy alone, suggesting that TNF may not be the dominant inflammatory driver in these patients. Instead, elevated epithelial IFN-I signaling may sustain a chronic inflammatory program that reinforces barrier dysfunction and suppresses mucosal healing. The restoration of epithelial function following IFN-I blockade supports a model in which IFN-I acts as a central regulator of anti-TNF non-response in NR-CD patients. Our results support a model in which TNF-dependent IFN-I signaling gates epithelial repair competence in CD, with metabolic or cytokine-targeted interventions effective only when this axis remains plastic. Future studies should test whether sustained IFN-I directly limits repair via stem cell fitness, metabolic wiring, or chromatin accessibility, and whether early reprogramming of this TNF–IFN-I axis can restore epithelial plasticity in NR-CDs.

A limitation of the current study is that we did not systematically test single agent treatments with anti-TNF or anti-IFN-I antibodies in all patient subgroups; rather, our experimental design prioritized combination therapies to model clinically relevant escalation strategies. In addition, the number of patient-derived organoid lines within each subgroup, particularly anti-TNF NR-CDs, was low. Although this reflects the practical and ethical constraints of obtaining well-phenotyped intestinal biopsies with matched clinical response data, the limited sample size reduces statistical power to detect more subtle donor-specific effects and may not fully capture the biological heterogeneity of CD across diverse populations. Furthermore, although biopsies were collected from the sigmoid colon and predominantly from macroscopically non-inflamed tissue, donors were not matched for disease activity, or drug levels, potentially contributing to inter-patient variability in epithelial IFN-I activation. Clinical non-response may reflect differences in drug exposure or immunogenicity rather than pharmacodynamic resistance, and the NR-CD cohort should therefore be interpreted as clinically defined non-responders. A major strength, however, lies in the consistency and biological relevance of the findings. We demonstrate across human datasets and murine epithelium that TNF signaling directly modulates epithelial IFN-I programs, identify sustained IFN-I gene expression as a feature of anti-TNF non-responsiveness, and show that epithelial repair and IFN-I suppression are mechanistically linked but donor-specific. Importantly, we reveal that anti-IFN-I-blockade restores proliferation and barrier-function in NR-CD epithelia, whereas butyrate selectively benefits responders, and that triple combination therapy differentially normalizes IFN-I and cytokine signatures by subgroup. These findings support a model in which epithelial metabolic responsiveness and IFN-I signaling define distinct therapeutic dependencies in CD. Our data provide a rationale for stratified epithelial targeting and therapeutic combinations to be selected on a per-patient basis.

## Supplementary Material

jjag140_Supplementary_Data

## Data Availability

The RNA-Seq dataset is available at NCBI Gene Expression Omnibus, identified by the accession number GSE201013. Data, analytic methods, and study materials will be made available from the corresponding author on reasonable request and as per ethical approvals.

## Conference presentations

Australian and New Zealand Society for Immunology, Perth, 2025; Digestive Diseases Week, Washington DC, 2024.

## References

[1] Haens GR , van Deventer S. 25 years of anti-TNF treatment for inflammatory bowel disease: lessons from the past and a look to the future. Gut. 2021;70:1396. 10.1136/gutjnl-2019-320022 33431575

[2] Lopez-Siles M , Martinez-Medina M, Busquets D, et al Mucosa-associated *Faecalibacterium prausnitzii* and *Escherichia coli* co-abundance can distinguish irritable bowel syndrome and inflammatory bowel disease phenotypes. Int J Med Microbiol. 2014;304:464-475. 10.1016/j.ijmm.2014.02.009 24713205

[3] Zhou Y , Xu ZZ, He Y, et al Gut microbiota offers universal biomarkers across ethnicity in inflammatory bowel disease diagnosis and infliximab response prediction. mSystems. 2018;3:e00188-17. 10.1128/mSystems.00188-17 PMC5790872 29404425

[4] Wang C , Gu Y, Chu Q, et al Gut microbiota and metabolites as predictors of biologics response in inflammatory bowel disease: A comprehensive systematic review. Microbiol Res. 2024;282:127660. 10.1016/j.micres.2024.127660 38442454

[5] Fagundes RR , Belt SC, Bakker BM, et al Beyond butyrate: microbial fiber metabolism supporting colonic epithelial homeostasis. Trends Microbiol. 2024;32:178-189. 10.1016/j.tim.2023.07.014 37596118

[6] Salvi PS , Cowles RA. Butyrate and the intestinal epithelium: modulation of proliferation and inflammation in homeostasis and disease. Cells. 2021;10:1775. 10.3390/cells10071775 PMC8304699 34359944

[7] Pott J , Hornef M. Innate immune signalling at the intestinal epithelium in homeostasis and disease. EMBO Rep. 2012;13:684-698. 10.1038/embor.2012.96 PMC3410395 22801555

[8] Recharla N , Geesala R, Shi XZ. Gut microbial metabolite butyrate and its therapeutic role in inflammatory bowel disease: a literature review. Nutrients. 2023;15:2275. 10.3390/nu15102275 PMC10221771 37242159

[9] Andreou NP , Legaki E, Gazouli M. Inflammatory bowel disease pathobiology: the role of the interferon signature. Ann Gastro­enterol. 2020;33:125-133. 10.20524/aog.2020.0457 PMC7049232 32127733

[10] Cohen RH , Colgan SP. Mucosal responses to type II interferon in IBD. Inflamm Bowel Dis. 2025;31:2584-2592. 10.1093/ibd/izaf143 PMC12455598 40682561

[11] Gazouli M. Type I and II interferon signatures can predict the response to anti-TNF agents in inflammatory bowel disease donors: involvement of the microbiota. Inflamm. Bowel Dis. 2020;26:1543-1553. 10.1093/ibd/izaa216 32812029

[12] van Baarsen LG , Wijbrandts CA, Rustenburg F, et al Regulation of IFN response gene activity during infliximab treatment in rheumatoid arthritis is associated with clinical response to treatment. Arthritis Res Ther. 2010;12:R11. 10.1186/ar2912 PMC2875639 20096109

[13] Friedrich M , Pohin M, Powrie F. Cytokine networks in the pathophysiology of inflammatory bowel disease. Immunity. 2019;50:992-1006. 10.1016/j.immuni.2019.03.017 30995511

[14] Stetson DB , Medzhitov R. Type I interferons in host defense. Immunity. 2006;25:373-381. 10.1016/j.immuni.2006.08.007 16979569

[15] Chemudupati M , Kenney AD, Smith AC, et al Butyrate reprograms expression of specific interferon-stimulated genes. J Virol. 2020;94:e00326-20. 10.1128/JVI.00326-20 PMC7394905 32461320

[16] d’Aldebert E , Quaranta M, Sébert M, et al Characterization of human colon organoids from inflammatory bowel disease patients. Front Cell Dev Biol. 2020;8:363. 10.3389/fcell.2020.00363 PMC7287042 32582690

[17] Angus HC , et al An autologous colonic organoid-derived monolayer model to study immune-bacterial interactions in Crohn’s disease donors. Clin Transl Immunol. 2022;11:e1407. 10.1002/cti2.1407 PMC9342672 35924188

[18] Gadeock S , et al Tumor necrosis factor signaling promotes differentiation of colonic deep crypt secretory cells. bioRxiv. 2025; 10.1101/2025.09.08.674949

[19] [dataset] * Arijs I, Li K, Toedter G, et al Mucosal gene signatures to predict response to infliximab in ulcerative colitis. Gut. 2009;58:1612-1619. 10.1136/gut.2009.178665 19700435

[20] [dataset] * Smillie CS, Biton M, Ordovas-Montanes J, et al Intra- and inter-cellular rewiring of the human colon during ulcerative colitis. Cell. 2019;178:714-730.e22. 10.1016/j.cell.2019.06.029 PMC6662628 31348891

[21] Sahoo D , Seita J, Bhattacharya D, et al A method of mining developmentally regulated genes using Boolean implications. Proc Natl Acad Sci USA. 2010;107:5732-5737. 10.1073/pnas.0913635107 PMC2851930 20231483

[22] VanDussen KL , et al Abnormal small intestinal epithelial microvilli in Crohn’s disease. Gastroenterology. 2018;155:815-828. 10.1053/j.gastro.2018.05.028 PMC6378688 29782846

[23] Noble CL , Abbas AR, Lees CW, et al Characterization of intestinal gene expression profiles in Crohn’s disease by genome-wide microarray analysis. Inflamm Bowel Dis. 2010;16:1717-1728. 10.1002/ibd.21263 20848455

[24] Verstockt B , Verstockt S, Veny M, et al Expression levels of 4 genes in colon tissue might be used to predict which donors will enter endoscopic remission after vedolizumab therapy for inflammatory bowel diseases. Clin Gastroenterol Hepatol. 2020;18:1142-1151.e10. 10.1016/j.cgh.2019.08.030 PMC7196933 31446181

[25] Thibault R , Blachier F, Darcy-Vrillon B, et al Butyrate utilization by the colonic mucosa in inflammatory bowel diseases: a transport deficiency. Inflamm Bowel Dis. 2010;16:684-695. 10.1002/ibd.21108 19774643

[26] Peloquin JM , Goel G, Kong L, et al Characterization of candidate genes in inflammatory bowel disease-associated risk loci. JCI Insight. 2016;1:e87899. 10.1172/jci.insight.87899 PMC5033062 27668286

[27] Palucka AK , Blanck JP, Bennett L, Pascual V, Banchereau J. Cross-regulation of TNF and IFN-alpha in autoimmune diseases. Proc Natl Acad Sci U S A. 2005;102:3372-3377. 10.1073/pnas.0408506102 PMC552921 15728381

[28] Jarry A , Malard F, Bou-Hanna C, et al Interferon-alpha promotes Th1 response and epithelial apoptosis via inflammasome activation in human intestinal mucosa. Cell Mol Gastroenterol Hepatol. 2017;3:72-81. 10.1016/j.jcmgh.2016.09.007 PMC5247398 28174758

[29] Huys L , et al Type I interferon drives TNF-induced lethal shock. J Exp Med. 2009;206:1873-1882. 10.1084/jem.20090213 PMC2737160 19687227

[30] Shalapour S , Deiser K, Kühl AA, et al Interleukin-7 links T lymphocyte and intestinal epithelial cell homeostasis. PLoS One. 2012;7:e31939. 10.1371/journal.pone.0031939 PMC3288069 22384106

[31] Gao Y , Dai J, Xu XP, Liu PF, Shi RH. Role of interferon alpha-inducible protein 6 in modulating the proliferation, apoptosis and senescence of oesophageal squamous cell carcinoma cells. J Physiol Pharmacol. 2023;74:3. 10.26402/jpp.2023.3.04 37661181

[32] Ruffenach G , Medzikovic L, Aryan L, et al Intestinal IFNα4 promotes diet-induced pulmonary hypertension. Respir Res. 2024;25:419. 10.1186/s12931-024-03046-z PMC11606228 39609844

[33] Ramos GP , Papadakis KA. Mechanisms of inflammatory bowel diseases. Mayo Clin Proc. 2019;94:155-165. 10.1016/j.mayocp.2018.09.013 PMC6386158 30611442

[34] Pedro MP , et al GPCR screening reveals that the metabolite receptor HCAR3 regulates epithelial proliferation, migration, and cellular respiration. J. Invest Dermatol. 2023;S0022-202X : 03134-2. 10.1016/j.jid.2023.12.002 PMC11116076 38103827

[35] Gadeock S , Kemp R, Sahoo D, et al Type I interferon targets as predictors of clinical non-response to anti-TNFs in IBD patients and their regulation of epithelial repair responses. Gastroenterology. 2024;166:S-1353.

[36] Yao Y , Richman L, Higgs BW, et al Neutralization of interferon-alpha/beta-inducible genes and downstream effect in a phase I trial of an anti-interferon-alpha monoclonal antibody in systemic lupus erythematosus. Arthritis Rheum. 2009;60:1785-1796. 10.1002/art.24557 19479852

[37] Conrad C , Di Domizio J, Mylonas A, et al TNF blockade induces a dysregulated type I interferon response without autoimmunity in paradoxical psoriasis. Nat Commun. 2018;9:25. 10.1038/s41467-017-02466-4 PMC5750213 29295985

[38] King MR , Kemp RA, Gadeock S. Type I interferons in inflammatory bowel diseases: balancing barrier integrity, repair and inflammation in the intestinal epithelium. Front Med (Lausanne). 2025;12:1713424. 10.3389/fmed.2025.1713424 PMC12685839 41377807

[39] Major J , Crotta S, Llorian M, et al Type I and III interferons disrupt epithelial repair during recovery from viral infection. Science. 2020;369:712-717. 10.1126/science.abc2061 PMC7292500 32527928

[40] Rauch I , Hainzl E, Rosebrock F, et al Type I interferons have opposing effects during the emergence and recovery phases of colitis. Eur J Immunol. 2014;44:2749-2760. 10.1002/eji.201344401 24975266

[41] Drougkas K , Skarlis C, Mavragani C. Type I interferons in systemic autoimmune rheumatic diseases: pathogenesis, clinical features and treatment options. Mediterr. J Rheumatol. 2024;35:365-380. 10.31138/mjr.270324.tis PMC11345602 39193187

[42] Sprooten J , Garg AD. Type I interferons and endoplasmic reticulum stress in health and disease. Int Rev Cell Mol Biol. 2020;350:63-118. 10.1016/bs.ircmb.2019.10.004 PMC7104985 32138904

[43] O’Carroll SM , Henkel FDR, O’Neill LAJ. Metabolic regulation of type I interferon production. Immunol Rev. 2024;323:276-287. 10.1111/imr.13318 38465724

[44] Ivanov AI , Parkos CA, Nusrat A. Cytoskeletal regulation of epithelial barrier function during inflammation. Am J Pathol. 2010;177:512-524. 10.2353/ajpath.2010.100168 PMC2913378 20581053

[45] Arumugam P , Saha K, Nighot P. Intestinal epithelial tight junction barrier regulation by novel pathways. Inflamm Bowel Dis. 2025;31:259-271. 10.1093/ibd/izae232 PMC12476983 39321109

[46] Shin W , Wu A, Min S, et al Spatiotemporal gradient and instability of Wnt induce heterogeneous growth and differentiation of human intestinal organoids. iScience. 2020;23:101372. 10.1016/j.isci.2020.101372 PMC7398973 32745985

[47] Janes KA , Gaudet S, Albeck JG, et al The response of human epithelial cells to TNF involves an inducible autocrine cascade. Cell. 2006;124:1225-1239. 10.1016/j.cell.2006.01.041 16564013

[48] Traves PG , Murray B, Campigotto F, Galien R, Meng A, Di Paolo JA. JAK selectivity and the implications for clinical inhibition of pharmacodynamic cytokine signalling by filgotinib, upadacitinib, tofacitinib and baricitinib. Ann Rheum Dis. 2021;80:865-875. 10.1136/annrheumdis-2020-219012 PMC8237188 33741556

[49] Lei H , Crawford MS, McCole DF. JAK-STAT pathway regulation of intestinal permeability: pathogenic roles and therapeutic opportunities in inflammatory bowel disease. Pharmaceuticals (Basel). 2021;14:840. 10.3390/ph14090840 PMC8466350 34577540

[50] Jamilloux Y , El Jammal T, Vuitton L, Gerfaud-Valentin M, Kerever S, Sève P. JAK inhibitors for the treatment of autoimmune and inflammatory diseases. Autoimmun Rev. 2019;18:102390. 10.1016/j.autrev.2019.102390 31520803

[51] Benoit P , Maguire D, Plavec I, Kocher H, Tovey M, Meyer F. A monoclonal antibody to recombinant human IFN-alpha receptor inhibits biologic activity of several species of human IFN-alpha, IFN-beta, and IFN-omega. Detection of heterogeneity of the cellular type I IFN receptor. J Immunol. 1993;150:707-716. 8423335

[52] Felten R , Scher F, Sagez F, Chasset F, Arnaud L. Spotlight on anifrolumab and its potential for the treatment of moderate-to-severe systemic lupus erythematosus: evidence to date. Drug Des Devel Ther. 2019;13:1535-1543. 10.2147/DDDT.S170969 PMC6514126 31190735

