# Supplementary material for: Targeting epithelial IFN-I signaling restores barrier integrity in anti-TNF refractory Crohn’s disease
Source: J Crohns Colitis. 2026 Sep 19;20(9):jjag140. doi: 10.1093/ecco-jcc/jjag140 (PMC13589608; doi:10.1093/ecco-jcc/jjag140)
Supplement: Supplementary file 1 [file jjag140_supplementary_data.zip › SG_JCC_SI 2026-FINAL PROOF.docx]

**Online supplemental table 1** Donor characteristics by group.

| Patient Code | Response Status* | Sex | Age | Histological Endoscopic Severity | Region of colon | Treatment (when biopsies were collected)** |
| --- | --- | --- | --- | --- | --- | --- |
| NR-CD01 | Primary non-responder | F | 31 | normal | Sigmoid | On Humira (low levels) |
| NR-CD02 | Primary non-responder | M | 32 | inflamed | Sigmoid | On Humira and steroids (stopped 4 days prior) |
| NR-CD03 | Primary non-responder | M | 23 | inflamed | Sigmoid | On Humira |
| NR-CD04 | Secondary non-responder | F | 54 | normal | Sigmoid | No medication after Humira |
| NR-CD05 | Secondary non-responder | M | 40 | normal | Sigmoid | Undetectable drug levels; IFX was stopped 2/12 prior |
| NR-CD06 | Secondary non-responder | F | 45 | normal | Sigmoid | On anti-IL23 after Humira |
| NR-CD07 | Secondary non-responder | F | 34 | normal | Sigmoid | On anti-TNF at the time |
| NR-CD08 | Secondary non-responder | F | 24 | normal | Sigmoid | to IFX; on Ustekinumab |
| R-CD01 | Responder | M | 29 | normal | Sigmoid | On IFX |
| R-CD02 | Responder | M | 21 | normal | Sigmoid | On Humira |
| R-CD03 | Responder | F | 48 | inflamed | Sigmoid | On Humira |
| R-CD04 | Responder | M | 28 | normal | Sigmoid | On Humira |
| R-CD05 | Responder | F | 55 | normal | Sigmoid | On Humira |
| R-CD06 | Responder | M | 33 | normal | Sigmoid | On Humira |
| R-CD07 | Responder | M | 33 | normal | Sigmoid | No medication |
| R-CD08 | Responder | M | 32 | normal | Sigmoid | On steroids and MTX |
| R-CD09 | Responder | F | 34 | normal | Sigmoid | On IFX |
| HC01 | non-IBD | F | 53 | N/A | Sigmoid | N/A |
| HC02 | non-IBD | F | 49 | N/A | Sigmoid | N/A |
| HC03 | non-IBD | F | 54 | N/A | Sigmoid | N/A |
| HC04 | non-IBD | F | 57 | N/A | Sigmoid | N/A |
| HC05 | non-IBD | F | 51 | N/A | Sigmoid | N/A |
| HC06 | non-IBD | F | 59 | N/A | Sigmoid | N/A |
| HC07 | non-IBD | F | 58 | N/A | Sigmoid | N/A |
| HC08 | non-IBD | F | 55 | N/A | Sigmoid | N/A |
| HC09 | non-IBD | F | 52 | N/A | Sigmoid | N/A |
| HC10 | non-IBD | F | 52 | N/A | Sigmoid | N/A |
| HC11 | non-IBD | F | 57 | N/A | Sigmoid | N/A |
| HC12 | non-IBD | F | 62 | N/A | Sigmoid | N/A |
| HC13 | non-IBD | M | 38 | N/A | Sigmoid | N/A |
| HC14 | non-IBD | M | 42 | N/A | Sigmoid | N/A |
| HC15 | non-IBD | M | 33 | N/A | Sigmoid | N/A |
| HC16 | non-IBD | F | 49 | N/A | Sigmoid | N/A |
| HC17 | non-IBD | F | 21 | N/A | Sigmoid | N/A |
| HC18 | non-IBD | F | 51 | N/A | Sigmoid | N/A |
| HC19 | non-IBD | M | 61 | N/A | Sigmoid | N/A |

*Response status refers to response to anti-TNF therapy at the time of sample collection. Primary non-responders failed to achieve an initial clinical response to anti-TNF therapy, whereas secondary non-responders initially responded but subsequently lost response. Clinical non-response was used as the inclusion criterion for the NR-CD group and was not assumed to represent pharmacodynamic resistance to anti-TNF therapy. **Treatment refers to therapy at the time of biopsy collection. Humira (adalimumab) and IFX (infliximab) are anti-TNF therapies; drug brand names may vary internationally. IL-23, interleukin-23 inhibitor; Ustekinumab, an IL-12/23 inhibitor; MTX, methotrexate. All samples were collected from the sigmoid colon. Summary data: NR-CD, N = 8, median age 33 years, 4F:4M; R-CD, N = 9, median age 33 years, 3F:6M; non-IBD controls, N = 19, median age 52 years, 14F:5M.

**Online supplemental table 2** Summary of external datasets used in this study, including dataset type and corresponding figure references where the data were analyzed.

| Reference | Dataset Description | | Associated Figures |
| --- | --- | --- | --- |
| Smillie et al. (2019)²³ | | Single cell RNA-Seq | figure 1A |
| GSE201013^21^ | | RNA-Seq | figure 1C, and  online supplemental figure 1A-C |
| Arijs et al. (2009)²² | | RNA-Seq | figure 1B, D and figure 3A |
| Van Dussen et al. (2018)^32^ | | RNA-Seq | online supplemental figure 2A |
| Noble et al. (2010)^34^ | | RNA-Seq | online supplemental figure 2B |
| Verstockt et al. (2020)^36^ | | RNA-Seq | online supplemental figure 2C |
| IBD cDNA Array (OriGene Technologies, Rockville, MA, USA; no. CCRT501) | | cDNA Array | online supplemental figure 1D |

**Online Supplemental Table 3** Distribution of responder Crohn’s disease, non-responder Crohn’s disease, and (non-IBD) control organoid lines across figures.

| **Donor ID** | **Group** | **Figure(s)** |
| --- | --- | --- |
| R-CD01 | Responder | Figure 2A, Figure 2B, Figure 2C, Supp. Figure 3A, Supp. Figure 3B, Supp. Figure 4A |
| R-CD02 | Responder | Figure 2A, Figure 2B, Figure 2C, Supp. Figure 3A, Supp. Figure 3B, Supp. Figure 3C, Supp. Figure 4A |
| R-CD03 | Responder | Figure 2B, Figure 2C, Supp. Figure 3A, Figure 3B, Supp. Figure 4A, Supp. Figure 4B, Supp. Figure 4E, Supp. Figure 4F |
| R-CD04 | Responder | Supp. Figure 3B, Supp. Figure 3C, Figure 3B, Figure 3C, Figure 3D, Supp. Figure 4A, Supp. Figure 4B, Supp. Figure 4E, Supp. Figure 4F, Figure 4A–E, Supp. Figure 5A–D, Figure 5A–E, Supp. Figure 6A–D |
| R-CD05 | Responder | Figure 3B, Figure 3C, Figure 3D, Supp. Figure 4B, Supp. Figure 4E, Supp. Figure 4F, Figure 4A–E, Supp. Figure 5A–D, Figure 5A–E, Supp. Figure 6A–D |
| R-CD06 | Responder | Figure 4A–E, Supp. Figure 5A–D, Figure 5A–E, Supp. Figure 6A–D |
| R-CD07 | Responder | Figure 4A–E, Supp. Figure 5A–D, Figure 5A–E, Supp. Figure 6A–D |
| R-CD08 | Responder | Figure 4A–E, Supp. Figure 5A–D, Figure 5A–E, Supp. Figure 6A–D |
| R-CD09 | Responder | Figure 4A–E, Supp. Figure 5A–D, Figure 5A–E, Supp. Figure 6A–D |
| NR-CD01 | Non-responder | Figure 2A, Figure 2B, Figure 2C, Supp. Figure 3A, Supp. Figure 3B, Supp. Figure 4A |
| NR-CD02 | Non-responder | Supp. Figure 3B, Supp. Figure 3C, Figure 3B, Figure 3C, Figure 3D, Supp. Figure 4A, Supp. Figure 4B, Supp. Figure 4E, Supp. Figure 4F |
| NR-CD03 | Non-responder | Supp. Figure 3B, Figure 3B, Figure 3D, Supp. Figure 4A, Supp. Figure 4E, Figure 4A–E, Supp. Figure 5A–D, Figure 5A–E, Supp. Figure 6A–D |
| NR-CD04 | Non-responder | Figure 3B, Figure 3C, Figure 3D, Supp. Figure 4A, Supp. Figure 4B, Supp. Figure 4E, Supp. Figure 4F, Figure 4A–E, Supp. Figure 5A–D, Figure 5A–E, Supp. Figure 6A–D |
| NR-CD05 | Non-responder | Figure 3C, Supp. Figure 4B, Supp. Figure 4F, Figure 4A–E, Supp. Figure 5A–D, Figure 5A–E, Supp. Figure 6A–D |
| NR-CD07 | Non-responder | Figure 4A–E, Supp. Figure 5A–D, Figure 5A–E, Supp. Figure 6A–D |
| NR-CD09 | Non-responder | Figure 4A–E, Supp. Figure 5A–D, Figure 5A–E, Supp. Figure 6A–D |
| HC01 | Non-IBD control | Figure 2A, Figure 2B, Figure 2C, Supp. Figure 3A, Supp. Figure 3B, Supp. Figure 3C, Figure 3B, Figure 3D, Supp. Figure 4A, Supp. Figure 4E |
| HC02 | Non-IBD control | Figure 2A, Supp. Figure 3A, Supp. Figure 3B, Figure 3B, Figure 3D, Supp. Figure 4E |
| HC03 | Non-IBD control | Figure 2A, Figure 2B, Supp. Figure 3A, Supp. Figure 3B, Supp. Figure 4A |
| HC04 | Non-IBD control | Figure 2B, Supp. Figure 3B, Supp. Figure 3C, Supp. Figure 4A |
| HC05 | Non-IBD control | Figure 2B, Figure 2C, Supp. Figure 3A, Supp. Figure 3B, Supp. Figure 3C, Supp. Figure 4A |
| HC06 | Non-IBD control | Figure 2B, Supp. Figure 3A, Supp. Figure 3B, Supp. Figure 3C, Supp. Figure 4A |
| HC07 | Non-IBD control | Supp. Figure 3B |
| HC08 | Non-IBD control | Supp. Figure 3B |
| HC09 | Non-IBD control | Figure 3B, Figure 3D, Supp. Figure 4E, Figure 4A–E, Supp. Figure 5A–D, Figure 5A–E, Supp. Figure 6A–D |
| HC10 | Non-IBD control | Figure 3C, Supp. Figure 4B, Supp. Figure 4F, Figure 4A–E, Supp. Figure 5A–D, Figure 5A–E, Supp. Figure 6A–D |
| HC11 | Non-IBD control | Figure 3C, Supp. Figure 4B, Supp. Figure 4F, Figure 4A–E, Supp. Figure 5A–D, Figure 5A–E, Supp. Figure 6A–D |
| HC12 | Non-IBD control | Figure 3C, Supp. Figure 4B, Supp. Figure 4F, Figure 4A–E, Supp. Figure 5A–D, Figure 5A–E, Supp. Figure 6A–D |
| HC13 | Non-IBD control | Optimisation experiments |
| HC14 | Non-IBD control | Optimisation experiments |
| HC15 | Non-IBD control | Optimisation experiments |
| HC16 | Non-IBD control | Figure 4A–E, Supp. Figure 5A–D, Figure 5A–E, Supp. Figure 6A–D |
| HC17 | Non-IBD control | Figure 4A–E, Supp. Figure 5A–D, Figure 5A–E, Supp. Figure 6A–D |
| HC18 | Non-IBD control | Optimisation experiments |
| HC19 | Non-IBD control | Optimisation experiments |

*HC13, HC14, HC15, HC18, HC19- optimization experiments for optimal antibody concentrations.

**Online supplemental figure 1** IFN-I driven transcriptional and pathway reprogramming in TNFR1 deficient intestinal epithelium and human IBD. A) Enrichment plots by Gene set enrichment analysis (GSEA) identified ‘INTERFERON ALPHA RESPONSE’, as the top 'Hallmark' (Molecular Signatures Database, MSigDB) gene sets enriched in TNFR1^-/-^ (Knockout: KO) colonic organoids profiled at baseline (unstimulated conditions) compared to TNFR1^+/-^ controls (WT), derived from the distal end of the colon of 16-week-old TNFR1^+/-^ and TNFR1^-/-^ mice. NES (Normalized enrichment score) versus p-value. B) Reactome pathway enrichment dot plot. Each dot represents an enriched Reactome pathway. The x-axis shows fold enrichment, and the y-axis lists pathway terms. Dot size corresponds to gene count, and dot colour indicates significance (-log10 Benjamini-Hochberg adjusted p-value), with darker colours reflecting higher significance. The schematic illustrates how genes enriched in the Reactome pathway “*Modulation of host responses”* by IFN-I stimulated genes function downstream. IFN-I stimulated genes cluster into coordinated modules regulating antiviral defense, interferon signaling and feedback control, antigen presentation, cytokine mediated immune modulation, and cell death pathways, highlighting the broad epithelial and immune regulatory impact of IFN I responses. C) A heatmap showing differential gene expression (DEG) of the IFN-I pathway between TNFR1^-/-^ (KO) colonic organoids compared to TNFR1^+/-^ controls (WT). Red and blue shadings represent higher and lower relative expression levels, as indicated respectively. C) The REACTOME pathway enrichment analysis of IFN-I pathway DEGs in TNFR1^-/-^ (KO) colonic organoids compared to TNFR1^+/-^ controls (WT). The significantly altered pathways are listed as per *p*-values. *N* = 3. Dataset used in this analysis is included in online supplemental table 2. D) A cDNA array analysis of IFN-I gene targets in the intestinal mucosa of CD donors relative to controls. *N* = 6, Controls; *N* = 21 NA-CD; *N* = 3 NR-CD and *N* = 7 R-CD donors. NA-CD denotes Crohn’s disease samples with unavailable anti-TNF response classification. Data was analyzed by one-way Anova, with a Šídák post-hoc test, where ****p < 0.0001, ***p < 0.001, **p < 0.01, *p < 0.05. Datasets used in these analyses are included in online supplemental table 2.

**Online supplemental figure 2** Differential IFN-I target gene expression before and after therapy in Crohn’s disease. Re-Analysis of single cell RNA Sequencing data showing IFN-I target genes enriched in the epithelium of CD donors before and after treatment with A) Ustekinumab, *N* = 26, Controls; *N* = 362, CD; *N* = 179 CD-Non-Responders (NR); and *N* = 183 CD-Responders B) Azathioprine, *N* = 73, Controls; *N* = 99, CD; *N* = 30 NR-CD; and *N* = 56 R-CD, and C) Vedolizumab, *N* = 43, CD; *N* = 25 NR-CD; and *N* = 18 R-CD. Data was analyzed by one-way Anova, with a Šídák post-hoc test where ****p < 0.0001, ***p < 0.001, **p < 0.01, *p < 0.05. Datasets used in these analyses are included in online supplemental table 2.

**Online supplemental figure 3** Butyrate alters epithelial permeability and stem and goblet cell marker expression in control and Crohn’s disease organoids. The effect of increasing concentrations of butyrate (1 mM and 10 mM) (72 h) exposure on A) the permeability of the epithelium, determined by translocation of FITC-Dextran 4K across the epithelium (*N* = 4-6) in organoids derived from control (non-IBD) and CD donors. Data were analyzed by a One-way Anova with a Šídák post-hoc test, where, # represents values normalized to untreated control. #- p < 0.05, ##-p < 0.01, ###-p < 0.001 and * represent comparisons between groups. *- p < 0.05, **-p < 0.01, ***-p < 0.001 and ****-p < 0.0001. Dotted line indicates the control at 1. B) Transcript levels of *Lgr5* and *Muc2* in organoids derived from control and CD donors, at baseline, as determined by qPCR. *N* = 9 Controls and *N* = 6 CD donors. Data was analyzed by an unpaired *t*-test, where ***p < 0.001. C) Transcript levels of *Lgr5* and *Muc2* in control and CD organoids exposed to 0.1 mM or 10 mM butyrate, as quantified by qPCR. *N* = 3 Controls and *N* = 3 CD donors. Data were analyzed by an unpaired *t*-test, where ***p < 0.001 and **p < 0.01. Donors included in these analyses are shown in online supplemental table 1 and -derived organoid lines included specifically in this analysis are listed in online supplementary table 3.

**Online supplemental figure 4** Epithelial responses to butyrate and damage in organoids and native primary tissue of Control (non-IBD) and CD patients. A) Transcript levels of butyrate receptors, *MCT1*, *GPR41*, *GPR43* and *HCAR3* in colonic organoids derived from control (non-IBD), NR-CD and R-CD organoids. *N* = 5-7 B) A representative image of an NR-CD organoid showing multi-lumen formation (white arrows) and apical-surface erosions (black arrows). Quantification of the number of multi-lumens formed in the epithelial layer of control, NR- and R-CD organoids exposed to CM ± 0.1 mM butyrate (72 h). *N* = 3. C) H&E-stained representative images of the intestinal mucosa from control, NR- and R-CD and UC donors, showing defects in epithelial structure and morphology in IBD donors. Black arrows indicate crypt abscess and loss. *N* = 6, Controls; *N* = 21, CD; and *N* = 21 UC; *N* = 3, NR-CD and *N* = 5, NR-UC. Scale bars = 250µm. Data were analyzed by a One-way Anova with a Šídák post-hoc test, where, # represents values normalized to untreated control. *- p < 0.05, ***-p < 0.001. Dotted line indicates control at 1. Donors included in these analyses are shown in online supplemental table 1 and -derived organoid lines included specifically in this analysis are listed in online supplementary table 3. H&E-stained images used in this analysis are part of the IBD cDNA array dataset, details included in online supplemental table 2. D) Table summarizing the statistical analysis of CM-induced changes, expressed relative to untreated controls, in control, NR-CD, and R-CD organoids (*determined from Figure 3D*). Data was analyzed by a One-way Anova with a Šídák post-hoc test, where *- p < 0.05, **-p < 0.01, ***-p < 0.001, ****-p < 0.0001. The effect of 50 ng/mL TNF, IL1β and IL6 on E) on immunolocalization and quantification of EdU, and CC3+ cells per organoid and F) permeability in organoids derived from control (non-IBD) and CD donors (R-Responder, NR-Non-Responder). Representative images show the localization of EdU+ (pink) and CC3+ (green) cells in nuclear-DAPI (blue) immunofluorescent organoids or FITC-Dextran 4K (green) in the basal and luminal compartment of the organoids. Scale bars = 200µm.

**Online supplemental figure 5** Butyrate and anti-IFN-I antibody treatment rescues loss in epithelial integrity and cytokine expression in a donor-specific manner for CD donors. The effect of 5 µg/mL anti-IFN-I antibody treatment ($\text{∝}$IFNα) on cytokine mix (CM) (48 h; 50 ng/mL TNF, IL1β and IL6) ± 0.1 mM Butyrate (72 h) exposure on A) EdU, and CC3+ cells per organoid in organoids derived from control (non-IBD) and CD donors (R-Responder, NR-Non-Responder). Representative images show the localization of EdU+ (pink) and CC3+ (green) cells in nuclear-DAPI (blue) immunofluorescent organoids. Scale bars = 200µm; B) on permeability of the epithelium as determined by translocation of FITC dextran across the epithelium in organoids derived from control (non-IBD) and NR- and R-CD donors. Representative images show the fluorescent FITC-Dextran 4K in the basal and luminal compartment of the organoids. *N* = 5-7. Scale bars = 200µm; C) mRNA levels of IFN-I targets in organoids derived from control and NR- and R-CD donors, quantified by qPCR. *N* = 5-7 and D) on cytokine levels quantified in the supernatants from organoids derived from control and NR- and R-CD donors, cytokine bead array (LEGENDplex™). Data were analyzed by a One-way Anova with a Kruskal-Wallis test, *- p < 0.05, **-p < 0.01, ***-p < 0.001, ****-p < 0.0001, respectively. Dotted line indicates data normalized to CM at 1. Donors included in these analyses are shown in online supplemental table 1 and -derived organoid lines included specifically in this analysis are listed in online supplementary table 3.

**Online supplemental figure 6** Butyrate and butyrate in combination with anti-TNF anti-IFN-I antibody treatment rescues loss in epithelial integrity and cytokine expression in a donor-specific manner for CD donors treated with anti-TNFs. The effect of 0.1 mM Butyrate (72 h) ± 5 µg/mL of anti-TNF ($\text{∝}$TNF) (Infliximab) or 0.1 mM Butyrate (72 h) ± 5 µg/mL of anti-TNF and 5 µg/mL anti-IFN-I antibody treatment on cytokine mix (CM) (48 h; 50 ng/mL TNF, IL1β, and IL6) was assessed on A) EdU, and CC3+ cells per organoid in organoids derived from control (non-IBD) and CD donors (R-Responder, NR-Non-Responder). Representative images show the localization of EdU+ (pink) and CC3+ (green) cells in nuclear-DAPI (blue) immunofluorescent organoids. Scale bars = 200µm; B) on permeability of the epithelium as determined by translocation of FITC dextran across the epithelium in organoids derived from control (non-IBD) and NR- and R-CD donors. Representative images show the fluorescent FITC-Dextran 4K in the basal and luminal compartment of the organoids. *N* = 5-7. Scale bars = 200µm; C) mRNA levels of IFN-I targets in organoids derived from control and NR- and R-CD donors, quantified by qPCR. *N* = 5-7 and D) on cytokine levels quantified in the supernatants from organoids derived from control and NR- and R-CD donors, cytokine bead array (LEGENDplex™). Data were analyzed by a One-way Anova with a Kruskal-Wallis test, *- p < 0.05, **-p < 0.01, ***-p < 0.001, ****-p < 0.0001, respectively. Dotted line indicates data normalized to CM at 1. Donors included in these analyses are shown in online supplemental table 1 and -derived organoid lines included specifically in this analysis are listed in online supplementary table 3.
